# Revisiting Artifacts of Kohn–Sham Density Functionals for Biosimulation

**DOI:** 10.1021/acs.jctc.4c00712

**Published:** 2024-07-31

**Authors:** Samuel A. Slattery, Jaden C. Yon, Edward F. Valeev

**Affiliations:** Department of Chemistry, Virginia Tech, Blacksburg, Virginia 24061, United States

## Abstract

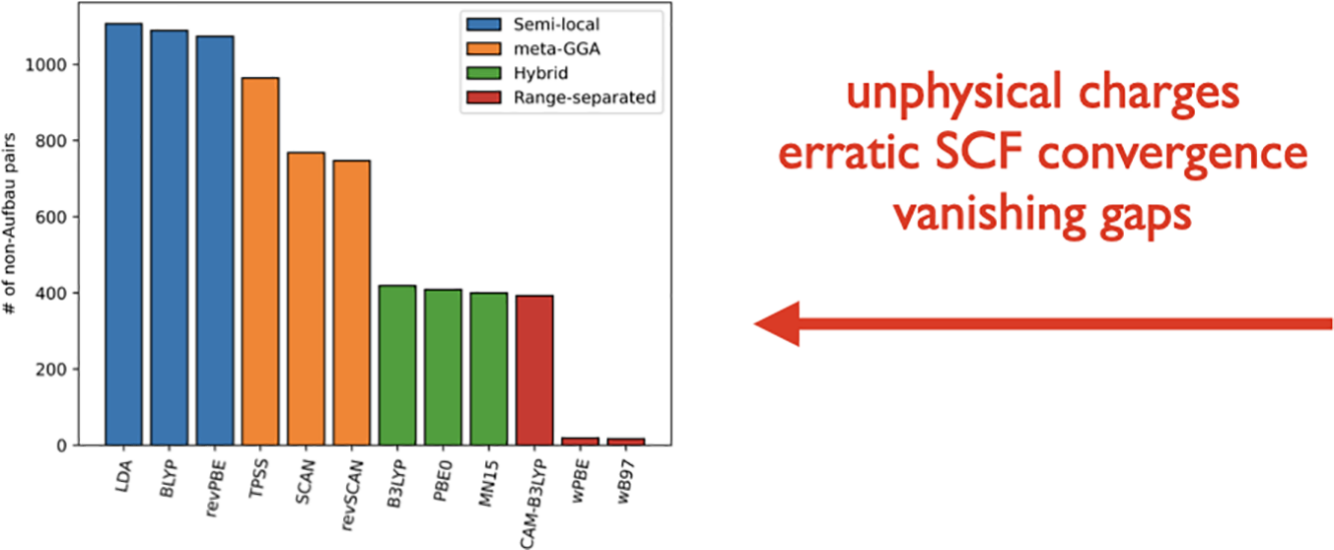

We revisit the problem of unphysical charge density delocalization/fractionalization induced by the self-interaction error of common approximate Kohn–Sham (KS) density functional theory functionals on simulation of small to medium-sized proteins in a vacuum. Aside from producing unphysical electron densities and total energies, the vanishing of the HOMO–LUMO gap associated with the unphysical charge delocalization leads to an unphysical low-energy spectrum and catastrophic failure of most popular solvers for the KS self-consistent field (SCF) problem. We apply a robust quasi-Newton SCF solver [Phys. Chem. Chem. Phys.2024, 26, 655738329140 ] to obtain solutions for some of these difficult cases. The anatomy of the charge delocalization is revealed by the *natural deformation orbitals* obtained from the density matrix difference between the Hartree–Fock and KS solutions; the charge delocalization not only can occur between charged fragments (such as in zwitterionic polypeptides) but also involves neutral fragments. The vanishing-gap phenomenon and troublesome SCF convergence are both attributed to the unphysical KS Fock operator eigenspectra of molecular fragments (e.g., amino acids or their side chains). Analysis of amino acid pairs suggests that the unphysical charge delocalization can be partially ameliorated by the use of *some* range-separated hybrid functionals but not by semilocal or standard hybrid functionals. Last, we demonstrate that solutions without the unphysical charge delocalization can be located even for semilocal KS functionals highly prone to such defects, but such solutions have non-Aufbau character and are unstable with respect to mixing of the non-overlapping “frontier” orbitals. Caution should be exercised when unexpectedly small (or vanishing) HOMO–LUMO gaps and atypical SCF convergence patterns (e.g., oscillatory) are observed in KS DFT simulations in any context (bio or otherwise).

## 1 Introduction

The unphysical behavior of all Kohn–Sham (KS) density functional approximations (DFAs) causes a number of artifacts, such as

- unrealistically small HOMO–LUMO gaps^1,2^ and excitation energies,^3^ especially for states with charge transfer^4^ and/or Rydberg^5^ character (although some dispute these notions^6^);
- charge fractionalization/delocalization, such as partial detachment of an electron in anions as the basis approaches completeness;^7,8^
- predicted reaction barriers that are too low;^9−13^
- incorrect dissociation curves for both cations^14^ and some neutrals;^15^
- overbinding of opposite-charged moieties,^16^ and charge transfer complexes.^17−19^

While these failures are well-recognized by the community, some artifactual behaviors of KS DFT in the context of biosimulations are less understood and invite more controversy. For example, it was discovered that SCF convergence problems appeared for some biomolecules when using semilocal DFAs in vacuum.^20−23^ Subsequent work concluded that no fundamental problem exists for semilocal DFAs when applied to such systems and that the problem was rather with the way the system was “prepared”.^24,25^ Ultimately this came down to two major factors: the missing solvent effects and geometry relaxation. Thus, commonly proposed solutions for the problem include (1) use of a solvent model (particularly implicit^25−27^ or even point charges^21^), (2) application of geometry relaxation,^24,25^ and (3) changing to a range-separated functional^23,28,29^ (e.g., ωB97). We agree that the problem is in the functional but note here that even range-separated functionals do not systematically solve these problems for all cases.^30^ The idea that the fundamental problem is electrostatic in nature rather than a result of more essential problems with the approximate functionals has persisted.^26,31^

Many researchers have realized that some of these problems can have striking consequences for the use of KS DFT in systems containing charged moieties. For example, Jensen^7^ recognized that the artificially high HOMO energy of the negatively charged deprotonated carboxylic acid group in a peptide zwitterion in vacuum could result in fractional charge transfer. While it has long been known that approximate KS DFT struggles with anions,^7,21,32−38^ the work of Jensen indicated the possibility of transfer of electron density within a single molecule. This possibility relates to the idea of “delocalization error”,^39−41^ where the density is too spread out due to the nonlinearity of the KS DFT total energy with respect to fractional electron number.^8,14,39,42,43^ Clearly, this can result in incorrect electrostatic descriptions,^24,44^ but it can also cause the aforementioned SCF convergence problems.^20,24^ One proposed solution to some of these issues involves evaluating KS DFT energies using HF densities,^37,38,45^ although the lack of self-consistency would then be a substantial concern.

In this work, we revisit the convergence problems of SCF for local and semilocal DFAs applied to medium-sized biomolecules in a vacuum. In section 3.1 we examine solutions for a set of 17 polypeptides considered in ref (21) using a robust local-convergence SCF solver (QUOTR^46^), thus allowing us to characterize the “true” (energy-minimized) solutions even for cases where no solutions could be found in ref (21); the SCF convergence issues are correlated to the unphysically small (or even vanishing) HOMO–LUMO gap. Although the incorrect density predicted by approximate functionals has been investigated for zwitterions,^27,44^ the orbital structure (at least for the semilocal functionals) was not examined in detail. Section 3.1 includes details of orbital-based analysis of KS → HF deformation densities revealing in pinpoint detail the anatomy of unphysical KS fractionalization/delocalization of charges in such systems. The use of deformation density to decompose a change in density into orbital contributions has precedent in the context of fragment to complex interaction,^47^ but it seems to be unknown for comparing different quantum chemistry methods. Additionally, we show that by using a local solver we can obtain SCF solutions with properly localized charges in some cases, which are actually non-Aufbau (and non-energy-minimizing). We follow this in section 3.2 by scanning through several popular functionals for all 20 naturally occurring amino acids to reveal which combinations of amino acids with which DFAs can cause such issues. In particular, we note that range separation, while almost always helpful, does not eliminate the possibility of unphysical charge delocalization and problematic SCF convergence.

## 2 Technical Details

HF and KS DFT computations on polypeptides in section 3.1 were performed with a developmental version of the Massively Parallel Quantum Chemistry (MPQC) version 4 program package^48^ using the recently developed QUOTR SCF solver.^46^ The maximum L-BFGS history size was set to 15 (parameter *m*), and the initial guess was the unperturbed version of the extended-Hückel-like guess used previously, except for 1RVS, which used the perturbed guess. The regularizer threshold (*t*_r_) was lowered to 0.15, and the history was also reset whenever the RMS gradient crossed 1 × 10^–6^ (either crossing below or coming back up again). However, the calculations on the glycine systems in section 3.2.1 used the same parameters as in ref (46). One final difference in the solver is that the history data were trimmed whenever the lowest eigenvalue of the matrix **V**^T^**V** was below −1 × 10^–15^ until that condition was no longer true. The KS DFT implementation in MPQC employs GauXC^49^ (which calls LibXC^50^) for calculation of the exchange–correlation potentials and energies. The integration grid for evaluation of these quantities for the PDB systems was the “superfine” grid (250 radial Mura–Knowles^51^ points and 974 angular Lebedev–Laikov points^52^ for all atoms except hydrogen, which has 175 radial points.). Density functionals used include LDA (SVWN5),^53,54^ BLYP,^55^ PBE,^56^ B3LYP,^57^ and PBE0^58,59^ for the PDB systems and, additionally, revPBE,^60^ MN15,^61^ TPSS,^62^ SCAN,^63^ revSCAN,^64^ CAM-B3LYP,^65^ ωB97,^66^ ωPBE^67,68^ for the peptide pair screening in section 3.2.2. To match the calculations performed in ref (21), we used the 6-31G** basis.^69−73^ Density fitting was performed with the def2-universal-J basis.^74^ Geometries for all systems (except the individual amino acids used in section 3.2) were obtained from the Protein Data Bank (PDB).^75^

The electron densities of the converged KS DFT solutions were analyzed by comparing them with the corresponding HF electron densities. This was done by finding the eigenvalues and eigenvectors of the difference of the density matrices (in an orthonormal basis):
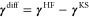
1These eigenvectors were then transformed back to the AO basis; these are the HF–KS *natural deformation orbitals* (NDOs). The eigenvalues associated with each NDO are termed natural deformation charges *q*_nd_. Plotting NDOs with negative *q*_nd_ identifies the regions where KS has gained density relative to HF, and vice versa. Although the use of a post-HF reference density would be preferred to include correlation effects (e.g., MP2), for the cases of qualitative failure of KS DFT the HF–KS and MP2–KS natural deformation orbitals should be qualitatively similar.

Amino acid calculations in section 3.2.2 used Psi4^76^ for both geometry optimization (B3LYP/6-31G*^69−73^) and single-point KS DFT energy evaluation (along with eigenspectrum) using default parameters, including density fitting. The side chains of the amino acids were uncharged in all cases, except for arginine, which had a +1 charge.

## 3 Results

### 3.1 Small Polypeptides

Our analysis starts with the set of 17 small polypeptides used by Rudberg to illustrate SCF convergence failures.^21^ For 12 of these systems, the standard diagonalization-based Roothaan–Hall (RH) SCF solver could not locate a solution for at least one functional; the chemical structures for these 12 “difficult” systems are presented in the Supporting Information. Using our QUOTR solver, we managed to obtain converged SCF solutions for all system/DFA combinations considered by Rudberg (the BHandHLYP functional was excluded since it posed no convergence issues). The HOMO–LUMO gaps of all fully converged solutions are reported in Table 1. For all RH-converged SCF solutions in ref (21), QUOTR confirmed the HOMO–LUMO gaps to within 0.04 eV (the largest deviation was observed for the 1XT7 system with the LDA functional). For the cases where the RH solver could not locate a solution, the QUOTR solver located solutions with vanishing HOMO–LUMO gap. These vanishing-gap QUOTR solutions were then analyzed by the HF–KS deformation density analysis (DDA) described in section 2.

**Table 1:** HOMO–LUMO Gaps (eV) for 17 Small Polypeptides from Reference (21)^a^

	HF		PBE0		B3LYP		BLYP		PBE		LDA	
PDB ID	RH	QUOTR	RH	QUOTR	RH	QUOTR	RH	QUOTR	RH	QUOTR	RH	QUOTR
2P7R	12.03	12.04	4.65	4.67	4.16	4.18	2.12	2.14	2.10	2.11	2.12	2.14
1BFZ	11.96	11.96	6.18	6.18	5.77	5.77	3.97	3.98	3.93	3.93	3.79	3.79
2IGZ	11.81	11.81	5.70	5.70	5.27	5.27	3.27	3.28	3.23	3.24	3.12	3.13
1D1E	10.14	10.12	3.96	3.96	3.47	3.47	1.56	1.57	1.58	1.59	1.54	1.55
1SP7	9.13	9.11	1.65	1.64	0.87	0.87	–	<0.01	–	<0.01	–	<0.01
1N9U	9.12	9.12	1.12	1.14	0.57	0.59	–	<0.01	–	<0.01	–	< 0.01
1MZI	8.77	8.74	1.29	1.29	0.54	0.54	–	<0.01	–	<0.01	–	<0.01
1XT7	8.51	8.48	3.32	3.29	2.65	2.63	1.02	1.00	1.24	1.23	1.30	1.34
1PLW	7.25	7.24	0.36	0.36	0.29	0.28	–	<0.01	–	<0.01	–	0.01
1FUL	6.95	6.93	0.20	0.20	0.16	0.16	–	–0.01	–	–0.01	–	–0.01
1EDW	6.89	6.90	0.26	0.26	0.21	0.21	–	<0.01	–	<0.01	–	<0.01
1EVC	5.82	5.84	0.30	0.30	0.24	0.24	–	<0.01	–	<0.01	–	<0.01
1RVS	5.60	5.59	–	0.10	–	0.08	–	<0.01	–	<0.01	–	<0.01
2FR9	5.48	5.50	0.26	0.26	0.21	0.21	–	<0.01	–	<0.01	–	<0.01
2JSI	5.26	5.27	0.24	0.24	0.19	0.19	–	<0.01	–	<0.01	–	<0.01
1LVZ	5.05	5.03	0.31	0.31	0.25	0.25	–	<0.01	–	< 0.01	–	<0.01
1FDF	3.64	3.62	0.13	0.14	0.11	0.11	–	<0.01	–	<0.01	–	<0.01

a The standard (RH) SCF solver values are from ref (21), whereas the quasi-Newton (QUOTR) SCF solver^46^ values are from this work. For all cases where the standard solver failed to converge to a solution, the quasi-Newton solver located a solution with a vanishing HOMO–LUMO gap.

#### 3.1.1 HF–LDA Deformation Density Analysis Summary

Although Hartree–Fock is known to artificially localize charges,^40^ it provides qualitatively correct electron distributions for all these systems; specifically, the HF charges agree with the expected integral values of formal charges on charged functional groups like −CO_2_^–^. In all cases, HF predicts large positive HOMO–LUMO gaps. In contrast, KS DFT can “delocalize” charges across large distances, or alternatively, it can lead to fractional charges on a charged functional group; such states have a vanishing HOMO–LUMO gap. The HF–KS DDA reveals where the fractional charges are located in the KS solution. The summary of HF–LDA DDA performed for the 12 systems with vanishing LDA gap (Table 2) reports which and how many functional groups donate (*N*_d_) and accept (*N*_a_) electron density in LDA relative to HF. Usually, but not always, the donor and acceptor sites correspond to the location of the Hartree–Fock HOMO and LUMO or other states near the Fermi level. See the Supporting Information for images comparing the Hartree–Fock HOMO and LUMO for each system with the natural deformation orbitals used in our analysis. In all cases shown in Table 2, the electron donors are formally negatively charged moieties (mostly −CO_2_^–^). The electron acceptors are often positively charged (mostly −NH_3_^+^); however, a few cases involve neutral functional groups as acceptors, most notably 2FR9, 1EDW, 1EVC, and 2JSI. Sometimes the natural deformation orbitals are not so clear to interpret. The column labeled “backbone?” is used to indicate when there are some density change contributions that are hard to classify which involve other parts of the system. In most cases, these contributions include carbonyl groups in the peptide bonds.

**Table 2:** HF–LDA Natural Deformation Density Analysis^a^ for the 12 Systems with SCF Convergence Difficulties from Reference (21), Indicating Donor Group Types and Acceptor Group Types^b^

PDB ID	|*q*_nd_|	*N*_d_	donor types	*N*_a_	acceptor types	backbone?
1SP7	0.211	1	–CO_2_^–^	1	–NH_3_^+^	minimal
1N9U	0.282	2	–CO_2_^–^	1	–NHC(NH_2_)_2_^+^	yes
1MZI	0.284	2	–CO_2_^–^	1	–NH_3_^+^	minimal
1PLW	0.379	1	–CO_2_^–^	1	–NH_3_^+^	some
1FUL	0.411	2	–CO_2_^–^	2	–NH_3_^+^, −NHC(NH_2_)_2_^+^	some; S_2_
1EDW	0.414	2	–CO_2_^–^	1	phenyl near – NH_3_^+^	some
1EVC	0.447	1	–CO^–^	1	phenyl	yes
1RVS	0.439	1	–CO_2_^–^	1	–NH_3_^+^ and phenyl	minimal
2FR9	0.566	2	–CO^–^, –NH^–^	2	phenyl, amide	yes; S_2_
2JSI	0.468	1	–NH^–^	1	phenol	yes
1LVZ	0.687	3	–CO_2_^–^	2	–NH_3_^+^, −NHC(NH_2_)_2_^+^	some
1FDF	0.564	2	–CO^–^, –PRO^–^	3	–NH_3_^+^, −NHC(NH_2_)_2_^+^, amide	yes

a *N*_d_ is the “number of donors”, and *N*_a_ is the “number of acceptors” (see the text for further details).

b –NHC(NH_2_)_2_^+^ is protonated guanidine; −PRO is proline.

Table 2 also characterizes the HF–LDA NDOs with non-negligible *q*_nd_; such orbitals are termed frontier NDOs (FNDOs). For all systems studied, the smallest *q*_nd_ was simply the negative of the largest one, and thus, we only display the largest |*q*_nd_| for each system. In many cases, there is a single pair of FNDOs, which typically corresponds to the case of electrons transferred between single functional groups. However, in some systems there are multiple FNDOs (e.g., 1FUL); in such cases we only display the largest |*q*_nd_|. In Table 2 we record only donor/acceptor groups contributing to the FNDOs with |*q*_nd_| ≥ 0.2. Only three systems had multiple |*q*_nd_| over this threshold: 1FUL, 2FR9, and 1FDF. Images of all natural deformation orbitals used in the analysis are provided in the Supporting Information.

We next examine three different cases that provide insight into the possible scenarios.

#### 3.1.2 Case 1FUL: Two Donors, Two Acceptors

**Figure 1:**
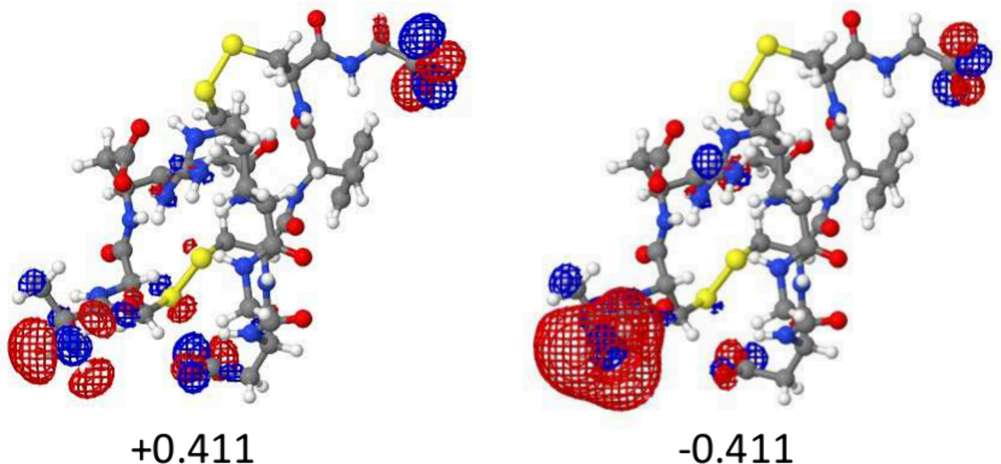
HF–LDA NDOs of 1FUL with largest-magnitude deformation charges. This case illustrates charge delocalization over multiple donor and acceptor functional groups.

First, we consider a zwitterion that exemplifies the classic case of charge separation, where the delocalization error is known to be a problem.^27,44^ In Figure 1 we display the natural deformation orbitals with the largest deformation charges, *q*_nd_ = ±0.411. In this case, there are actually at least two regions that appear to be accepting electrons: the NH_3_^+^ group and the protonated guanidine group of arginine. Also, there are two deprotonated carboxylic acid groups that donate electrons. Although this case is very similar to the classic zwitterions, the analysis is made more complicated because there is density transfer occurring with the peptide backbone and at least one of the disulfide bridges.

#### 3.1.3 Case 1EVC: Neutral Acceptor

**Figure 2:**
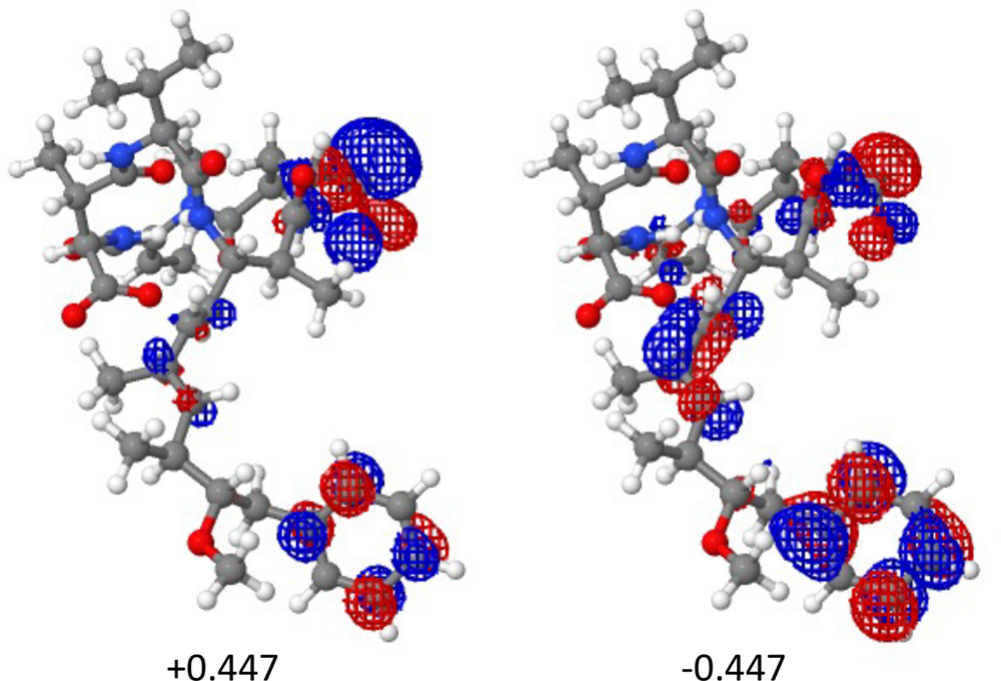
HF–LDA natural deformation orbitals of 1EVC with the largest-magnitude deformation charges.

The problems with zwitterion convergence using semilocal DFAs are relatively well known. Here we show that it is possible to have the same problem without an explicitly positive group receiving the electron density. In Figure 2 the natural deformation orbitals of the largest magnitude are displayed for 1EVC. Closer examination of the structure reveals that electron density is being donated from a CO^–^ (not CO_2_^–^) group! Clearly this system has an invalid structure generated to fit the experimental NMR data. For such unphysical structures, it is possible to have an extremely unstable anion whose density will be transferred to a neutral group. However, such exceptions are rare, since this is one of the few examples of anion-to-neutral charge transfer in the current test set.

#### 3.1.4 Case 1RVS: Non-Aufbau Solution

**Figure 3:**
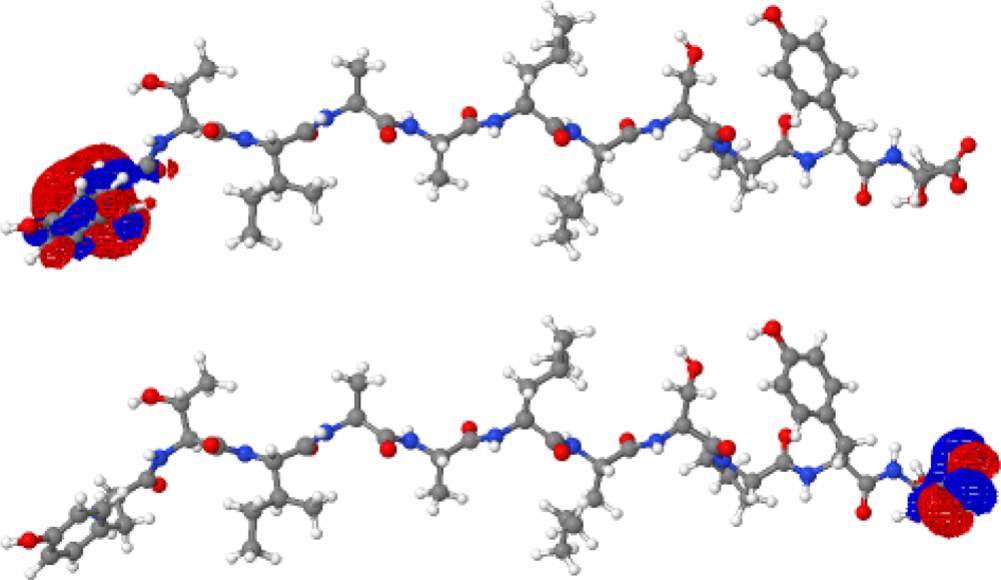
For the 1RVS zwitterion, it was possible to locate a non-Aufbau B3LYP SCF soluton that does not suffer from charge delocalization and qualitatively matches the charge distribution of the exact ground state. The non-Aufbau HOMO (bottom) and LUMO (top) are localized on the CO_2_^–^ and NH_3_^+^ termini, as expected.

Although the near-zero gap displayed for 1FUL in section 3.1.2 is typical for zwitterions in vacuum, we have found that for a different zwitterion our local SCF solver is able to converge to the “physically correct” charge-separated solution. In Figure 3 we display the HOMO and LUMO for 1RVS when the QUOTR solver is given the usual extended-Hückel-like initial guess for the orbitals, but without perturbation.^46^ We see that there is no unphysical mixing of the occupied orbital on the CO_2_^–^ with the unoccupied orbital on the NH_3_^+^. For this solution, the HOMO has an energy of −0.037 eV while the LUMO has an energy of −0.130 eV, giving a nonzero negative HOMO–LUMO gap. Thus, this is a non-Aufbau state! Now, this solution is not the best solution in a variational sense, because the total energy could be lowered by mixing the HOMO with the LUMO (note Janak’s theorem^77^). However, it does avoid the major contribution to the incorrect charge delocalization. We conclude that we have been able to find a local stationary point for 1RVS (a zwitterion system) using B3LYP that has qualitatively the physically correct HOMO and LUMO, but such a solution is not the lowest-energy state. When the initial guess is perturbed, QUOTR can converge to the nearly zero-gap solution. The energy of this solution is 0.012 *E*_h_ below the physically reasonable non-Aufbau solution.

### 3.2 Amino Acids

The simplest analogous situation where the HOMO could be above the LUMO is in a system composed of two amino acids (in vacuum) separated by a large distance so that they are essentially noninteracting. This simple model is explored in section 3.2.1, where we show that the non-Aufbau solution is obtainable using the QUOTR SCF solver. By perturbing the guess orbitals (importantly mixing orbitals on the separated fragments), we can also obtain the near-zero-gap solution. The underlying principle thus indicates that this will occur whenever the HOMO of one fragment is above the LUMO of the other fragment. Therefore, in section 3.2.2 we calculate HOMO and LUMO energies for a variety of popular functionals to see which combinations are possibly problematic.

#### 3.2.1 Separated Glycine Zwitterion

We demonstrate the utility of QUOTR by finding a solution for a system that is impossible for a diagonalization-based solver: a non-Aufbau-filled system. For this analysis, we use KS-DFT with the local density approximation (LDA) to the exchange–correlation functional.

**Figure 4:**
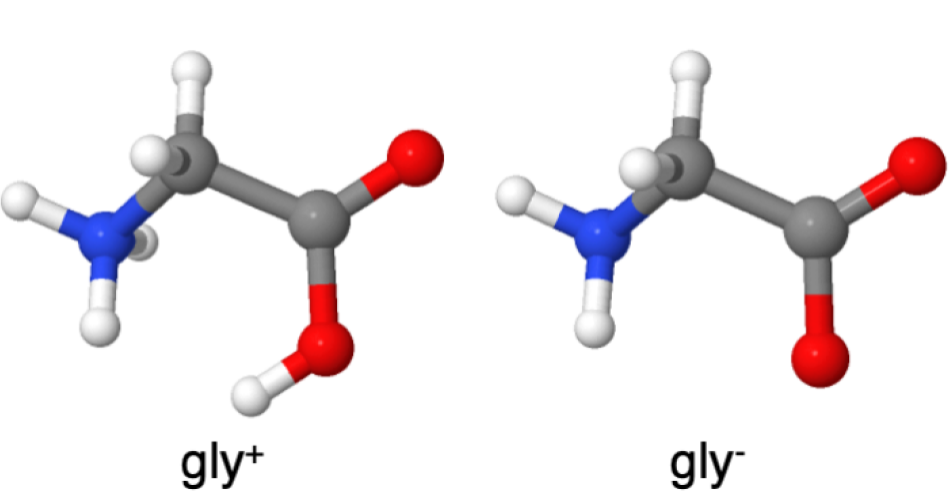
Glycine in protonated (overall positive charge, gly^+^) and deprotonated (overall negative charge, gly^–^) states.

The HOMO and LUMO for glycine in two protonation states (labled gly^–^ and gly^+^; see Figure 4) were calculated separately. These could represent the two ends of a peptide chain in zwitterion form. Then a supersystem was constructed with these two fragments together, separated by approximately 200 Å. Thus, the two fragments should be physically isolated from each other.

From the data in Table 3 we see that a non-Aufbau solution is expected: although each charged fragment has a sizable positive HOMO–LUMO gap, the HOMO of the anion is much higher than the LUMO of the cation. When these two fragments are calculated together, the initial guess for the MOs is of the utmost importance for any direct minimization solver. In the column labeled “unperturbed”, the initial guess is our standard extended-Hückel-like guess. Due to the large separation of about 200 Å, there is no overlap between the AOs of the different fragments. Thus, the guess MOs are disjoint: each orbital is either associated with gly^–^ or gly^+^. The gradient for mixing the MOs on different fragments should therefore be zero. Minimization should then result in nearly the same orbitals on each fragment as in the isolated calculations, implying a solution with the HOMO higher than the LUMO! In contrast, the column labeled “perturbed” takes the extended-Hückel-like guess and applies a small, random unitary matrix that allows mixing between all orbitals, regardless of the fragment to which they belong. Thus, there are likely some orbitals in the initial guess that span both fragments. This is physically incorrect, but LDA is actually able to find a lower total energy for the supersystem with some MOs spanning both fragments. The HOMO is then lowered and the LUMO raised until the gap is essentially zero.

**Table 3:** Frontier Orbital Energetics (eV) of LDA/6-31G** SCF Solutions for Isolated Deprotonated and Protonated Glycine Molecules and Their Noninteracting Supersystem^a^

			gly^–^···gly^+^	
	gly^–^	gly^+^	unperturbed	perturbed
LUMO	5.72	–6.79	–6.72	–3.31
HOMO	0.64	–12.59	0.57	–3.29
HOMO–LUMO gap	5.08	5.80	–7.29	–0.02

a The quasi-Newton solver locates a non-Aufbau supersystem solution unless the initial (atomic-density-based) guess is perturbed to mix the orbitals of the monomers. The perturbed guess leads to the solution with a vanishing HOMO–LUMO gap and an unphysical charge delocalization.

This striking example is due to the self-interaction error in the LDA functional, and it demonstrates LDA’s tendency to prefer fractional-electron systems. In this case, the fractional electron on one of the fragments is caused by having an MO with substantial density on the other fragment too.

#### 3.2.2 Screening Natural Amino Acid Pairs

To systematically investigate when the problem of the vanishing HOMO–LUMO gap is likely to appear, we do not need to run the same calculations as in the previous section for each pair of amino acids. The principle is clear: when an isolated fragment has a HOMO above the LUMO of a separate fragment, a non-Aufbau-filled solution is possible. The idea that having the LUMO on one fragment above the HOMO on another other fragment can cause excessive electron transfer and binding strength has been examined before in the context of radical–molecule complexes^78^ and charge transfer complexes.^79^ We performed a series of tests on different functionals for all 20 naturally occurring amino acids in three different protonation states: neutral, anionic, and cationic (except for arginine, where the charges are shifted to 0, +1, and +2). Table 4 displays the numbers of predicted non-Aufbau pairs of amino acids, grouped by charge state, for a representative set of local gradient-corrected (GGA), meta-GGA, and hybrid DFAs (both standard and range-separated).

**Table 4:** Numbers of Non-Aufbau States Predicted by Various KS Functionals for Noninteracting Pairs of the 20 Natural Amino Acids in Their Neutral/Protonated/Deprotonated States^a^

Semilocal																	
	LDA						BLYP						revPBE				
			*Q*_a_						*Q*_a_						*Q*_a_		
		–1	0	+1	+2			–1	0	+1	+2			–1	0	+1	+2
*Q*_d_	–1	0	379	380	19	*Q*_d_	–1	0	380	380	19	*Q*_d_	–1	0	380	380	19
	0	0	0	306	20		0	0	0	286	20		0	0	0	273	20
	+1	0	0	0	3		+1	0	0	0	4		+1	0	0	0	2
	+2	0	0	0	0		+2	0	0	0	0		+2	0	0	0	0

Meta-GGA																	
	TPSS						SCAN						revSCAN				
			*Q*_a_						*Q*_a_						*Q*_a_		
		–1	0	+1	+2			–1	0	+1	+2			–1	0	+1	+2
*Q*_d_	–1	0	368	380	19	*Q*_d_	–1	0	275	380	19	*Q*_d_	–1	0	265	380	19
	0	0	0	176	20		0	0	0	74	20		0	0	0	63	20
	+1	0	0	0	1		+1	0	0	0	0		+1	0	0	0	0
	+2	0	0	0	0		+2	0	0	0	0		+2	0	0	0	0

Hybrid																	
	MN15^b^						B3LYP						PBE0				
			*Q*_a_						*Q*_a_						*Q*_a_		
		–1	0	+1	+2			–1	0	+1	+2			–1	0	+1	+2
*Q*_d_	–1	0	0	380	19	*Q*_d_	–1	0	0	380	19	*Q*_d_	–1	0	0	380	19
	0	0	0	0	1		0	0	0	0	20		0	0	0	0	10
	+1	0	0	0	0		+1	0	0	0	0		+1	0	0	0	0
	+2	0	0	0	0		+2	0	0	0	0		+2	0	0	0	0

Range-Separated																	
	CAM-B3LYP						ωB97						ωPBE				
			*Q*_a_						*Q*_a_						*Q*_a_		
		–1	0	+1	+2			–1	0	+1	+2			–1	0	+1	+2
*Q*_d_	–1	0	0	374	19	*Q*_d_	–1	0	0	0	17	*Q*_d_	–1	0	0	0	19
	0	0	0	0	0		0	0	0	0	0		0	0	0	0	0
	+1	0	0	0	0		+1	0	0	0	0		+1	0	0	0	0
	+2	0	0	0	0		+2	0	0	0	0		+2	0	0	0	0

a The data are broken down by charges *Q*_d_ and *Q*_a_ of the “donor” and “acceptor” fragments, respectively (i.e., containing the HOMO and LUMO, respectively).

b MN15 is both a hybrid functional and a meta functional, but we list it under “Hybrid”.

**Figure 5:**
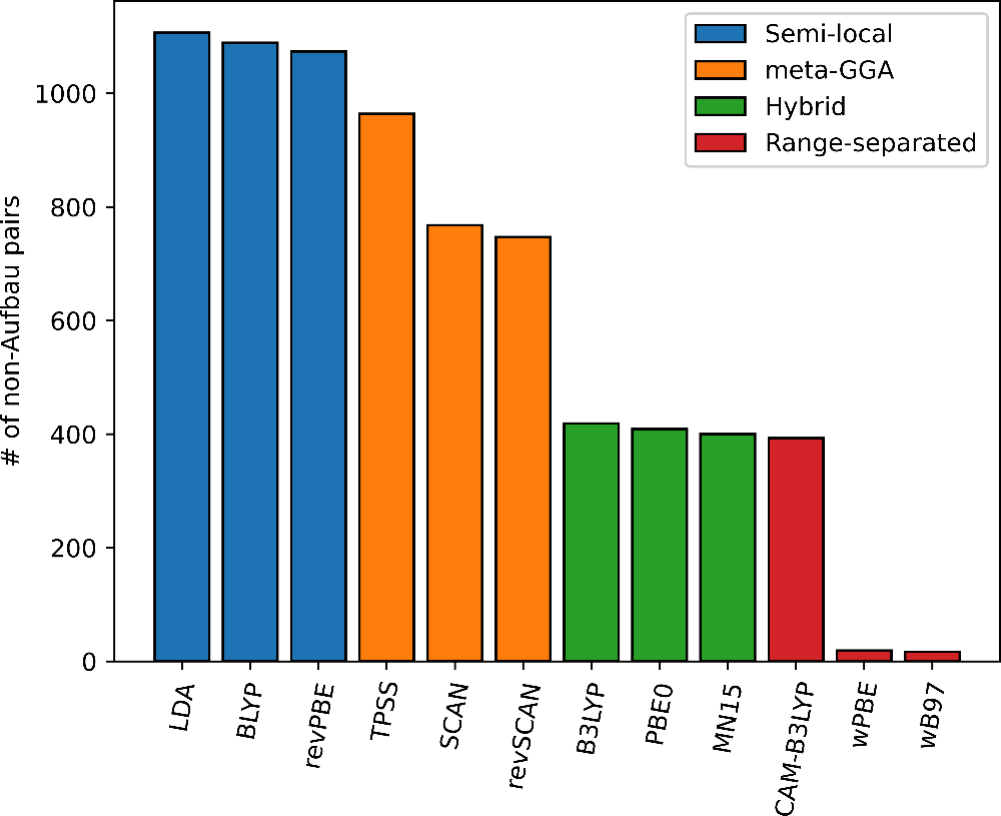
Overall numbers of non-Aufbau states predicted by various KS functionals for noninteracting pairs of the 20 natural amino acids in their neutral/protonated/deprotonated states. See Table 4 for the further breakdown by the amino acid charges.

As expected, including larger fractions of the Hartree–Fock exchange reduces the number of problematic combinations (Figure 5). The performance of the semilocal functionals is almost unchanged going from LDA to GGA. The meta-GGAs provide some improvement but not as much improvement as hybrid functionals. Finally, the range-separated functionals perform best of all; however, there are some cases where they still predict non-Aufbau-filled systems. The most difficult systems are when the donor is negatively charged and the acceptor has a +2 charge (arginine), where even ωB97 fails.

## 4 Summary

This work reexamined the SCF convergence problems for polypeptides in the gas phase in conjunction with modern nonhybrid and hybrid DFAs. While standard SCF solvers typically fail catastrophically,^21,27^ using a robust quasi-Newton SCF solver^46^ we were able to obtain SCF solutions when the conventional solvers fail; in such cases, the KS solutions always had a vanishing HOMO–LUMO gap. Deeper analysis of these solutions using novel natural deformation orbitals obtained from the HF–KS density matrix difference reveals which regions of the system donate and accept electron density in the unphysical KS DFT solution and are thus the culprits in the delocalization error. In general, the unphysical charge delocalization can involve not only charged moieties but also formally neutral fragments (e.g., a phenyl ring), demonstrating that zwitterions are not the only problematic cases for semilocal DFAs. The origin of the unphysical charge delocalization is the misalignment of the KS Fock operator eigenspectrum between molecular fragments. A systematic study of pairs of 20 naturally occurring amino acids in various protonation states suggested that the unphysical charge delocalization is only partially reduced by the use of a more sophisticated functional. The range-separated functionals employing 100% exact exchange at long range were a nearly perfect remedy, albeit not totally immune to the problem. The rest of the representative functional families (standard hybrid, meta-GGA, GGA) all suffered from unphysical charge delocalization to various extents.

The most direct lesson from our work is the need for caution when unexpectedly small (or vanishing) HOMO–LUMO gaps and atypical SCF convergence patterns (e.g., oscillatory) are observed in KS DFT simulations in any context (bio or otherwise). Such anomalies should call for probing the solution (e.g., population analysis) and testing more advanced KS functionals. Although our work focused on a specific, and somewhat artificial, biosimulation context (namely, isolated polypeptides), such systems continue to serve as components and even as the sole focus of benchmark datasets^80^ for training more approximate models. There are also lessons here for a broader class of Kohn–Sham DFT simulations. As an affordable source of first-principles potentials for training more approximate models, KS DFT is increasingly used to generate massive datasets for benchmarking and training purposes,^81^ with scale too large for validatation of even a non-negligible portion of the dataset. Thus, as the role of KS DFT as the “ground truth” model rises, so should our expectations of its accuracy and robustness. Although we can expect that the continuing improvement of KS functionals will reduce the occurrence of the artifacts discussed here, the need for robust solvers will continue to increase with the degree of automation of KS DFT-based workflows.

## Supporting Information Available

Geometries of the 12 polypeptides with “difficult” SCF convergence considered in section 3.1 and images juxtaposing the Hartree–Fock HOMO and LUMO of each system with the corresponding HF–LDA natural deformation orbitals (PDF)

## References

[ref1] Tsuneda T.; Song J.-W.; Suzuki S.; Hirao K. On Koopmans’ Theorem in Density Functional Theory. J. Chem. Phys. 2010, 133, 174101 10.1063/1.3491272. 21054000

[ref2] Rudberg E.; Rubensson E. H.; Sałek P. Kohn-Sham Density Functional Theory Electronic Structure Calculations with Linearly Scaling Computational Time and Memory Usage. J. Chem. Theory Comput. 2011, 7, 340–350. 10.1021/ct100611z. 26596156

[ref3] Tozer D. J. Relationship between Long-Range Charge-Transfer Excitation Energy Error and Integer Discontinuity in Kohn-Sham Theory. J. Chem. Phys. 2003, 119, 12697–12699. 10.1063/1.1633756.

[ref4] Dreuw A.; Weisman J. L.; Head-Gordon M. Long-Range Charge-Transfer Excited States in Time-Dependent Density Functional Theory Require Non-Local Exchange. J. Chem. Phys. 2003, 119, 2943–2946. 10.1063/1.1590951.

[ref5] Casida M. E.; Jamorski C.; Casida K. C.; Salahub D. R. Molecular Excitation Energies to High-Lying Bound States from Time-Dependent Density-Functional Response Theory: Characterization and Correction of the Time-Dependent Local Density Approximation Ionization Threshold. J. Chem. Phys. 1998, 108, 4439–4449. 10.1063/1.475855.

[ref6] Baerends E. J.; Gritsenko O. V.; Van Meer R. The Kohn-Sham Gap, the Fundamental Gap and the Optical Gap: The Physical Meaning of Occupied and Virtual Kohn-Sham Orbital Energies. Phys. Chem. Chem. Phys. 2013, 15, 16408 10.1039/c3cp52547c. 24002107

[ref7] Jensen F. Describing Anions by Density Functional Theory: Fractional Electron Affinity. J. Chem. Theory Comput. 2010, 6, 2726–2735. 10.1021/ct1003324. 26616074

[ref8] Peach M. J. G.; Teale A. M.; Helgaker T.; Tozer D. J. Fractional Electron Loss in Approximate DFT and Hartree-Fock Theory. J. Chem. Theory Comput. 2015, 11, 5262–5268. 10.1021/acs.jctc.5b00804. 26574320

[ref9] Dobbs K. D.; Dixon D. A. Ab Initio Prediction of the Activation Energy for the Abstraction of a Hydrogen Atom from Methane by Chlorine Atom. J. Phys. Chem. 1994, 98, 12584–12589. 10.1021/j100099a021.

[ref10] Johnson B. G.; Gonzales C. A.; Gill P. M.; Pople J. A. A Density Functional Study of the Simplest Hydrogen Abstraction Reaction. Effect of Self-Interaction Correction. Chem. Phys. Lett. 1994, 221, 100–108. 10.1016/0009-2614(94)87024-1.

[ref11] Zhang Q.; Bell R.; Truong T. N. Ab Initio and Density Functional Theory Studies of Proton Transfer Reactions in Multiple Hydrogen Bond Systems. J. Phys. Chem. 1995, 99, 592–599. 10.1021/j100002a022.

[ref12] Vydrov O. A.; Scuseria G. E. A Simple Method to Selectively Scale down the Self-Interaction Correction. J. Chem. Phys. 2006, 124, 191101 10.1063/1.2204599. 16729796

[ref13] Janesko B. G.; Scuseria G. E. Hartree-Fock Orbitals Significantly Improve the Reaction Barrier Heights Predicted by Semilocal Density Functionals. J. Chem. Phys. 2008, 128, 244112 10.1063/1.2940738. 18601322 PMC2809668

[ref14] Zhang Y.; Yang W. A Challenge for Density Functionals: Self-interaction Error Increases for Systems with a Noninteger Number of Electrons. J. Chem. Phys. 1998, 109, 2604–2608. 10.1063/1.476859.

[ref15] Dutoi A. D.; Head-Gordon M. Self-Interaction Error of Local Density Functionals for Alkali-Halide Dissociation. Chem. Phys. Lett. 2006, 422, 230–233. 10.1016/j.cplett.2006.02.025.

[ref16] Otero-de-la-Roza A.; Johnson E. R. Analysis of Density-Functional Errors for Noncovalent Interactions between Charged Molecules. J. Phys. Chem. A 2020, 124, 353–361. 10.1021/acs.jpca.9b10257. 31846333

[ref17] Ruiz E.; Salahub D. R.; Vela A. Charge-Transfer Complexes: Stringent Tests for Widely Used Density Functionals. J. Phys. Chem. 1996, 100, 12265–12276. 10.1021/jp9533077.

[ref18] Isborn C. M.; Mar B. D.; Curchod B. F. E.; Tavernelli I.; Martínez T. J. The Charge Transfer Problem in Density Functional Theory Calculations of Aqueously Solvated Molecules. J. Phys. Chem. B 2013, 117, 12189–12201. 10.1021/jp4058274. 23964865

[ref19] Otero-de-la-Roza A.; Johnson E. R.; DiLabio G. A. Halogen Bonding from Dispersion-Corrected Density-Functional Theory: The Role of Delocalization Error. J. Chem. Theory Comput. 2014, 10, 5436–5447. 10.1021/ct500899h. 26583227

[ref20] Rubensson E. H.; Rudberg E. Bringing about Matrix Sparsity in Linear-scaling Electronic Structure Calculations. J. Comput. Chem. 2011, 32, 1411–1423. 10.1002/jcc.21723. 21284001

[ref21] Rudberg E. Difficulties in Applying Pure Kohn-Sham Density Functional Theory Electronic Structure Methods to Protein Molecules. J. Phys.: Condens. Matter 2012, 24, 072202 10.1088/0953-8984/24/7/072202. 22223667

[ref22] Antony J.; Grimme S. Fully Ab Initio Protein-ligand Interaction Energies with Dispersion Corrected Density Functional Theory. J. Comput. Chem. 2012, 33, 1730–1739. 10.1002/jcc.23004. 22570225

[ref23] Kulik H. J.; Luehr N.; Ufimtsev I. S.; Martinez T. J. Ab Initio Quantum Chemistry for Protein Structures. J. Phys. Chem. B 2012, 116, 12501–12509. 10.1021/jp307741u. 22974088

[ref24] Lever G.; Cole D. J.; Hine N. D. M.; Haynes P. D.; Payne M. C. Electrostatic Considerations Affecting the Calculated HOMO-LUMO Gap in Protein Molecules. J. Phys.: Condens. Matter 2013, 25, 152101 10.1088/0953-8984/25/15/152101. 23470878

[ref25] Cole D. J.; Hine N. D. M. Applications of Large-Scale Density Functional Theory in Biology. J. Phys.: Condens. Matter 2016, 28, 393001 10.1088/0953-8984/28/39/393001. 27494095

[ref26] Zuehlsdorff T. J.; Haynes P. D.; Hanke F.; Payne M. C.; Hine N. D. M. Solvent Effects on Electronic Excitations of an Organic Chromophore. J. Chem. Theory Comput. 2016, 12, 1853–1861. 10.1021/acs.jctc.5b01014. 26967019

[ref27] Ren F.; Liu F. Impacts of Polarizable Continuum Models on the SCF Convergence and DFT Delocalization Error of Large Molecules. J. Chem. Phys. 2022, 157, 184106 10.1063/5.0121991. 36379768

[ref28] Sepunaru L.; Refaely-Abramson S.; Lovrinčić R.; Gavrilov Y.; Agrawal P.; Levy Y.; Kronik L.; Pecht I.; Sheves M.; Cahen D. Electronic Transport via Homopeptides: The Role of Side Chains and Secondary Structure. J. Am. Chem. Soc. 2015, 137, 9617–9626. 10.1021/jacs.5b03933. 26149234

[ref29] Sharley J. N. Amino Acid Preference against Beta Sheet through Allowing Backbone Hydration Enabled by the Presence of Cation. arXiv (Physics.Chemical Physics), October 3, 2016, 1610.00375, ver. 1. https://arxiv.org/abs/1610.00375.

[ref30] Vydrov O. A.; Scuseria G. E. Assessment of a Long-Range Corrected Hybrid Functional. J. Chem. Phys. 2006, 125, 234109 10.1063/1.2409292. 17190549

[ref31] Li J.-H.; Zuehlsdorff T. J.; Payne M. C.; Hine N. D. M. Identifying and Tracing Potential Energy Surfaces of Electronic Excitations with Specific Character via Their Transition Origins: Application to Oxirane. Phys. Chem. Chem. Phys. 2015, 17, 12065–12079. 10.1039/C5CP01018G. 25875632

[ref32] Shore H. B.; Rose J. H.; Zaremba E. Failure of the Local Exchange Approximation in the Evaluation of the H^–^ Ground State. Phys. Rev. B 1977, 15, 2858–2861. 10.1103/PhysRevB.15.2858.

[ref33] Schwarz K. First Ionisation Potentials of Atoms Obtained with Local-Density Schemes. J. Phys. B: At. Mol. Phys. 1978, 11, 1339–1351. 10.1088/0022-3700/11/8/007.

[ref34] Schwarz K. Instability of Stable Negative Ions in the Xα Method or Other Local Density Functional Schemes. Chem. Phys. Lett. 1978, 57, 605–607. 10.1016/0009-2614(78)85330-5.

[ref35] Rösch N.; Trickey S. B. Comment on “Concerning the Applicability of Density Functional Methods to Atomic and Molecular Negative Ions” [J. Chem. Phys. 105, 862 (1996)]. J. Chem. Phys. 1997, 106, 8940–8941. 10.1063/1.473946.

[ref36] Peach M. J. G.; De Proft F.; Tozer D. J. Negative Electron Affinities from DFT: Fluorination of Ethylene. J. Phys. Chem. Lett. 2010, 1, 2826–2831. 10.1021/jz101052q.

[ref37] Lee D.; Furche F.; Burke K. Accuracy of Electron Affinities of Atoms in Approximate Density Functional Theory. J. Phys. Chem. Lett. 2010, 1, 2124–2129. 10.1021/jz1007033.

[ref38] Kim M.-C.; Sim E.; Burke K. Communication: Avoiding Unbound Anions in Density Functional Calculations. J. Chem. Phys. 2011, 134, 171103 10.1063/1.3590364. 21548663

[ref39] Mori-Sánchez P.; Cohen A. J.; Yang W. Many-Electron Self-Interaction Error in Approximate Density Functionals. J. Chem. Phys. 2006, 125, 201102 10.1063/1.2403848. 17144681

[ref40] Mori-Sánchez P.; Cohen A. J.; Yang W. Localization and Delocalization Errors in Density Functional Theory and Implications for Band-Gap Prediction. Phys. Rev. Lett. 2008, 100, 146401 10.1103/PhysRevLett.100.146401. 18518055

[ref41] Cohen A. J.; Mori-Sánchez P.; Yang W. Insights into Current Limitations of Density Functional Theory. Science 2008, 321, 792–794. 10.1126/science.1158722. 18687952

[ref42] Perdew J. P.; Parr R. G.; Levy M.; Balduz J. L. Density-Functional Theory for Fractional Particle Number: Derivative Discontinuities of the Energy. Phys. Rev. Lett. 1982, 49, 1691–1694. 10.1103/PhysRevLett.49.1691.

[ref43] Hait D.; Head-Gordon M. Delocalization Errors in Density Functional Theory Are Essentially Quadratic in Fractional Occupation Number. J. Phys. Chem. Lett. 2018, 9, 6280–6288. 10.1021/acs.jpclett.8b02417. 30339010

[ref44] Jakobsen S.; Kristensen K.; Jensen F. Electrostatic Potential of Insulin: Exploring the Limitations of Density Functional Theory and Force Field Methods. J. Chem. Theory Comput. 2013, 9, 3978–3985. 10.1021/ct400452f. 26592393

[ref45] Sim E.; Song S.; Vuckovic S.; Burke K. Improving Results by Improving Densities: Density-Corrected Density Functional Theory. J. Am. Chem. Soc. 2022, 144, 6625–6639. 10.1021/jacs.1c11506. 35380807

[ref46] Slattery S. A.; Surjuse K. A.; Peterson C. C.; Penchoff D. A.; Valeev E. F. Economical Quasi-Newton Unitary Optimization of Electronic Orbitals. Phys. Chem. Chem. Phys. 2024, 26, 6557–6573. 10.1039/D3CP05557D. 38329140

[ref47] Pakiari A. H.; Fakhraee S.; Azami S. M. Decomposition of Deformation Density into Orbital Components. Int. J. Quantum Chem. 2008, 108, 415–422. 10.1002/qua.21453.

[ref48] Peng C.; Lewis C. A.; Wang X.; Clement M. C.; Pierce K.; Rishi V.; Pavošević F.; Slattery S.; Zhang J.; Teke N.; Kumar A.; Masteran C.; Asadchev A.; Calvin J. A.; Valeev E. F. Massively Parallel Quantum Chemistry: A High-Performance Research Platform for Electronic Structure. J. Chem. Phys. 2020, 153, 044120 10.1063/5.0005889. 32752656

[ref49] Petrone A.; Williams-Young D. B.; Sun S.; Stetina T. F.; Li X. An Efficient Implementation of Two-Component Relativistic Density Functional Theory with Torque-Free Auxiliary Variables. Eur. Phys. J. B 2018, 91, 169 10.1140/epjb/e2018-90170-1.

[ref50] Lehtola S.; Steigemann C.; Oliveira M. J.; Marques M. A. Recent Developments in Libxc – A Comprehensive Library of Functionals for Density Functional Theory. SoftwareX 2018, 7, 1–5. 10.1016/j.softx.2017.11.002.

[ref51] Mura M. E.; Knowles P. J. Improved Radial Grids for Quadrature in Molecular Density-Functional Calculations. J. Chem. Phys. 1996, 104, 9848–9858. 10.1063/1.471749.

[ref52] Lebedev V. I.; Laikov D. N. A Quadrature Formula for the Sphere of the 131st Algebraic Order of Accuracy. Dokl. Math. 1999, 59, 477–481.

[ref53] Dirac P. A. M. Note on Exchange Phenomena in the Thomas Atom. Math. Proc. Cambridge Philos. Soc. 1930, 26, 376–385. 10.1017/S0305004100016108.

[ref54] Vosko S. H.; Wilk L.; Nusair M. Accurate Spin-Dependent Electron Liquid Correlation Energies for Local Spin Density Calculations: A Critical Analysis. Can. J. Phys. 1980, 58, 1200–1211. 10.1139/p80-159.

[ref55] Miehlich B.; Savin A.; Stoll H.; Preuss H. Results Obtained with the Correlation Energy Density Functionals of Becke and Lee, Yang and Parr. Chem. Phys. Lett. 1989, 157, 200–206. 10.1016/0009-2614(89)87234-3.

[ref56] Perdew J. P.; Burke K.; Ernzerhof M. Generalized Gradient Approximation Made Simple. Phys. Rev. Lett. 1996, 77, 3865–3868. 10.1103/PhysRevLett.77.3865. 10062328

[ref57] Stephens P. J.; Devlin F. J.; Chabalowski C. F.; Frisch M. J. Initio Calculation of Vibrational Absorption and Circular Dichroism Spectra Using Density Functional Force Fields. J. Phys. Chem. 1994, 98, 11623–11627. 10.1021/j100096a001.

[ref58] Adamo C.; Barone V. Toward Reliable Density Functional Methods without Adjustable Parameters: The PBE0 Model. J. Chem. Phys. 1999, 110, 6158–6170. 10.1063/1.478522.

[ref59] Ernzerhof M.; Scuseria G. E. Assessment of the Perdew-Burke-Ernzerhof Exchange-Correlation Functional. J. Chem. Phys. 1999, 110, 5029–5036. 10.1063/1.478401. 15268348

[ref60] Zhang Y.; Yang W. Comment on “Generalized Gradient Approximation Made Simple”. Phys. Rev. Lett. 1998, 80, 890–890. 10.1103/PhysRevLett.80.890.

[ref61] Yu H. S.; He X.; Li S. L.; Truhlar D. G. MN15: A Kohn-Sham Global-Hybrid Exchange-Correlation Density Functional with Broad Accuracy for Multi-Reference and Single-Reference Systems and Noncovalent Interactions. Chem. Sci. 2016, 7, 5032–5051. 10.1039/C6SC00705H. 30155154 PMC6018516

[ref62] Tao J.; Perdew J. P.; Staroverov V. N.; Scuseria G. E. Climbing the Density Functional Ladder: Nonempirical Meta-Generalized Gradient Approximation Designed for Molecules and Solids. Phys. Rev. Lett. 2003, 91, 146401 10.1103/PhysRevLett.91.146401. 14611541

[ref63] Sun J.; Ruzsinszky A.; Perdew J. P. Strongly Constrained and Appropriately Normed Semilocal Density Functional. Phys. Rev. Lett. 2015, 115, 036402 10.1103/PhysRevLett.115.036402. 26230809

[ref64] Mezei P. D.; Csonka G. I.; Kállay M. Simple Modifications of the SCAN Meta-Generalized Gradient Approximation Functional. J. Chem. Theory Comput. 2018, 14, 2469–2479. 10.1021/acs.jctc.8b00072. 29565589

[ref65] Yanai T.; Tew D. P.; Handy N. C. A New Hybrid Exchange-Correlation Functional Using the Coulomb-attenuating Method (CAM-B3LYP). Chem. Phys. Lett. 2004, 393, 51–57. 10.1016/j.cplett.2004.06.011.

[ref66] Chai J.-D.; Head-Gordon M. Systematic Optimization of Long-Range Corrected Hybrid Density Functionals. J. Chem. Phys. 2008, 128, 084106 10.1063/1.2834918. 18315032

[ref67] Henderson T. M.; Janesko B. G.; Scuseria G. E. Generalized Gradient Approximation Model Exchange Holes for Range-Separated Hybrids. J. Chem. Phys. 2008, 128, 194105 10.1063/1.2921797. 18500854 PMC2812874

[ref68] Weintraub E.; Henderson T. M.; Scuseria G. E. Long-Range-Corrected Hybrids Based on a New Model Exchange Hole. J. Chem. Theory Comput. 2009, 5, 754–762. 10.1021/ct800530u. 26609580

[ref69] Ditchfield R.; Hehre W. J.; Pople J. A. Self-Consistent Molecular-Orbital Methods. IX. An Extended Gaussian-Type Basis for Molecular-Orbital Studies of Organic Molecules. J. Chem. Phys. 1971, 54, 724–728. 10.1063/1.1674902.

[ref70] Hehre W. J.; Ditchfield R.; Pople J. A. Self—Consistent Molecular Orbital Methods. XII. Further Extensions of Gaussian—Type Basis Sets for Use in Molecular Orbital Studies of Organic Molecules. J. Chem. Phys. 1972, 56, 2257–2261. 10.1063/1.1677527.

[ref71] Hariharan P. C.; Pople J. A. The Influence of Polarization Functions on Molecular Orbital Hydrogenation Energies. Theor. Chim. Acta 1973, 28, 213–222. 10.1007/BF00533485.

[ref72] Francl M. M.; Pietro W. J.; Hehre W. J.; Binkley J. S.; Gordon M. S.; DeFrees D. J.; Pople J. A. Self-consistent Molecular Orbital Methods. XXIII. A Polarization-type Basis Set for Second-row Elements. J. Chem. Phys. 1982, 77, 3654–3665. 10.1063/1.444267.

[ref73] Gordon M. S.; Binkley J. S.; Pople J. A.; Pietro W. J.; Hehre W. J. Self-Consistent Molecular-Orbital Methods. 22. Small Split-Valence Basis Sets for Second-Row Elements. J. Am. Chem. Soc. 1982, 104, 2797–2803. 10.1021/ja00374a017.

[ref74] Weigend F. Accurate Coulomb-fitting Basis Sets for H to Rn. Phys. Chem. Chem. Phys. 2006, 8, 1057 10.1039/b515623h. 16633586

[ref75] Berman H. M. The Protein Data Bank. Nucleic Acids Res. 2000, 28, 235–242. 10.1093/nar/28.1.235. 10592235 PMC102472

[ref76] Smith D. G. A.; Burns L. A.; Simmonett A. C.; Parrish R. M.; Schieber M. C.; Galvelis R.; Kraus P.; Kruse H.; Di Remigio R.; Alenaizan A.; James A. M.; Lehtola S.; Misiewicz J. P.; Scheurer M.; Shaw R. A.; Schriber J. B.; Xie Y.; Glick Z. L.; Sirianni D. A.; O’Brien J. S.; Waldrop J. M.; Kumar A.; Hohenstein E. G.; Pritchard B. P.; Brooks B. R.; SchaeferIII H. F.; Sokolov A. Y.; Patkowski K.; DePrinceIII A. E.; Bozkaya U.; King R. A.; Evangelista F. A.; Turney J. M.; Crawford T. D.; Sherrill C. D. PSI4 1.4: Open-source Software for High-Throughput Quantum Chemistry. J. Chem. Phys. 2020, 152, 184108 10.1063/5.0006002. 32414239 PMC7228781

[ref77] Janak J. F. Proof That ∂*E*/∂*n*_*i*_ = ε in Density-Functional Theory. Phys. Rev. B 1978, 18, 7165–7168. 10.1103/PhysRevB.18.7165.

[ref78] Johnson E. R.; Salamone M.; Bietti M.; DiLabio G. A. Modeling Noncovalent Radical-Molecule Interactions Using Conventional Density-Functional Theory: Beware Erroneous Charge Transfer. J. Phys. Chem. A 2013, 117, 947–952. 10.1021/jp3084309. 23323943

[ref79] Sini G.; Sears J. S.; Brédas J.-L. Evaluating the Performance of DFT Functionals in Assessing the Interaction Energy and Ground-State Charge Transfer of Donor/Acceptor Complexes: Tetrathiafulvalene-Tetracyanoquinodimethane (TTF-TCNQ) as a Model Case. J. Chem. Theory Comput. 2011, 7, 602–609. 10.1021/ct1005517. 26596294

[ref80] Prasad V. K.; Otero-de-la-Roza A.; DiLabio G. A. PEPCONF, a Diverse Data Set of Peptide Conformational Energies. Sci. Data 2019, 6, 180310 10.1038/sdata.2018.310. 30667382 PMC6343515

[ref81] Culka M.; Kalvoda T.; Gutten O.; Rulíšek L. Mapping Conformational Space of All 8000 Tripeptides by Quantum Chemical Methods: What Strain Is Affordable within Folded Protein Chains?. J. Phys. Chem. B 2021, 125, 58–69. 10.1021/acs.jpcb.0c09251. 33393778

