# Supplementary material for: Revisiting Artifacts of Kohn–Sham Density Functionals for Biosimulation
Source: J Chem Theory Comput. 2024 Jul 31;20(15):6652–60. doi: 10.1021/acs.jctc.4c00712 (PMC11325537; doi:10.1021/acs.jctc.4c00712)
Supplement: Supplementary file 1 — ct4c00712_si_001.pdf [file ct4c00712_si_001.pdf]

Supporting Information: Revisiting Artifacts of Kohn-Sham Density Functionals for Biosimulation

Samuel A. Slattery, Jaden C. Yon, and Edward F. Valeev*

Department of Chemistry, Virginia Tech, Blacksburg, VA 24061

E-mail: efv@vt.edu

1 Structures of Systems from the PDB

Chemical structures of the 12 systems investigated in section 3.1 are presented here.

2 Natural Deformation Orbitals: Systems from the PDB using LDA

Images of the HF-LDA natural deformation orbitals with magnitudes of deformation charges greater than 0.2 ("Frontier Natural Deformation Orbitals", or FNDO) are presented in this section, along with the Hartree-Fock HOMO and LUMO.

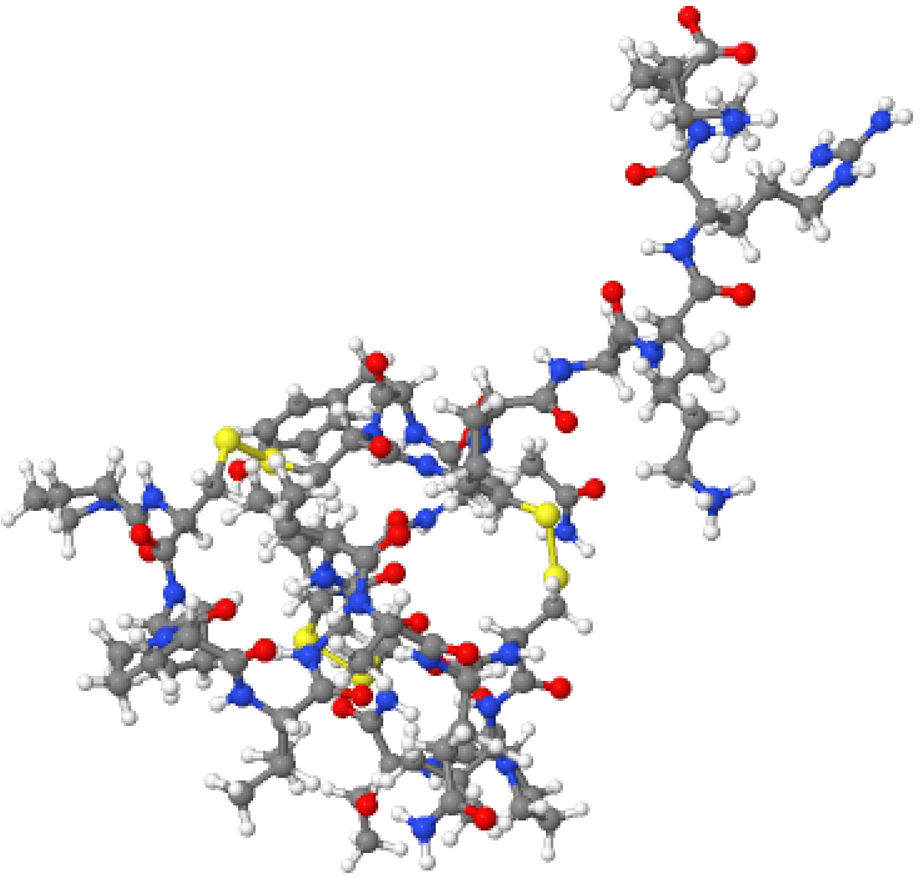

Figure S1: 1SP7

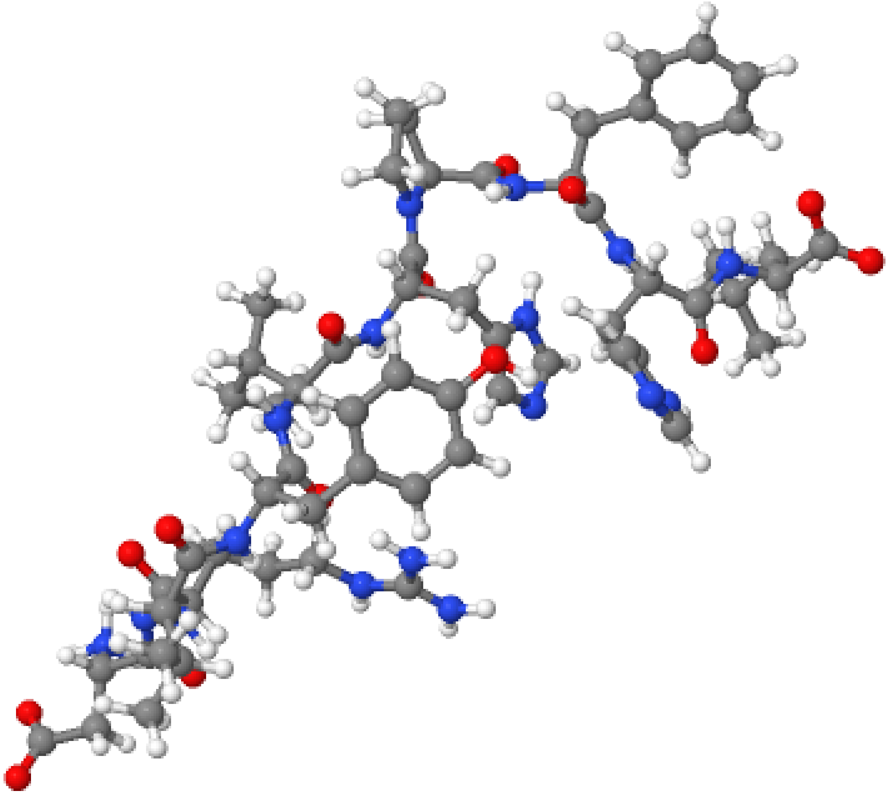

Figure S2: 1N9U

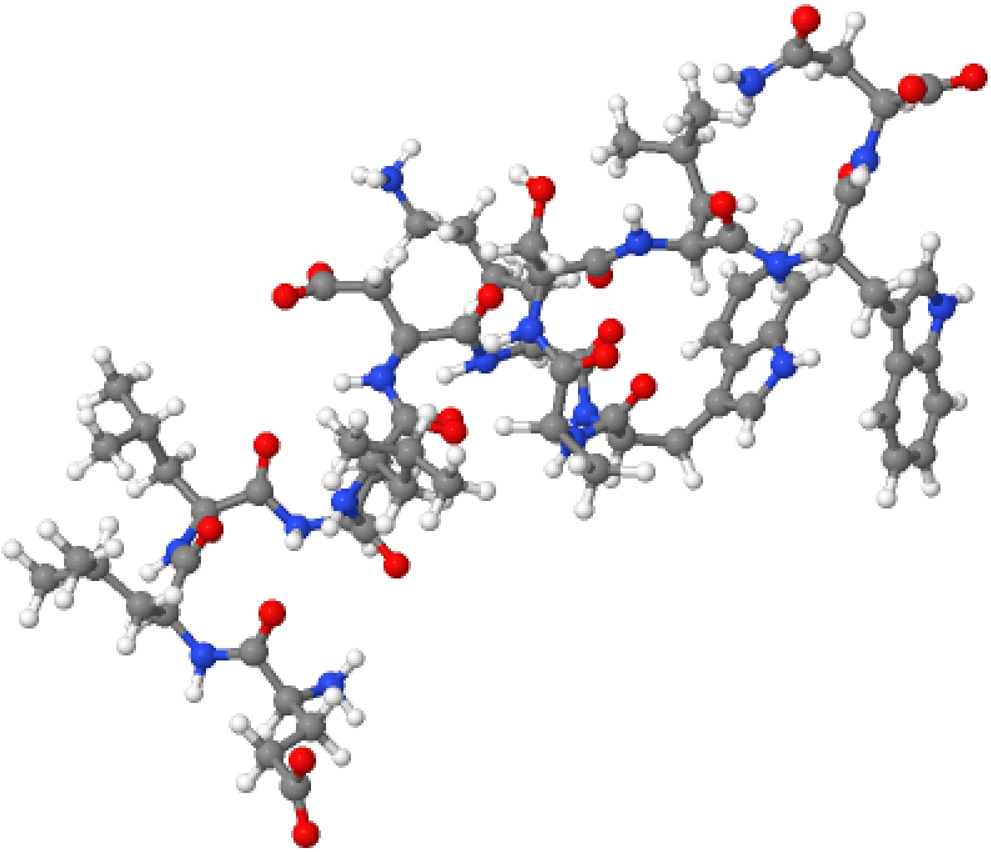

Figure S3: 1MZI

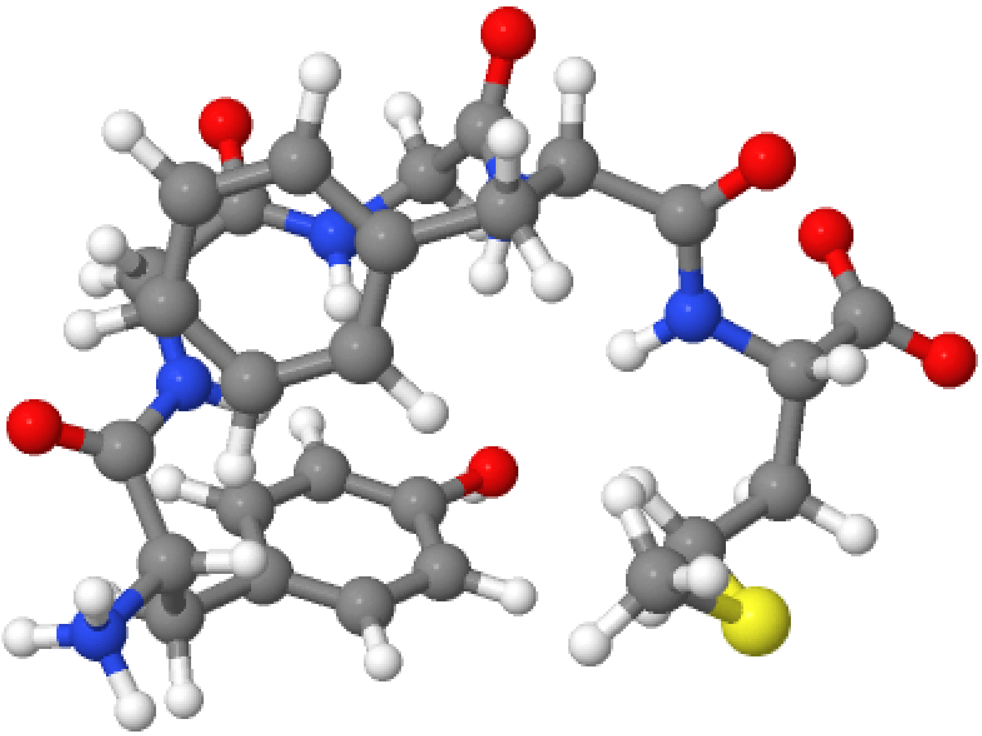

Figure S4: 1PLW

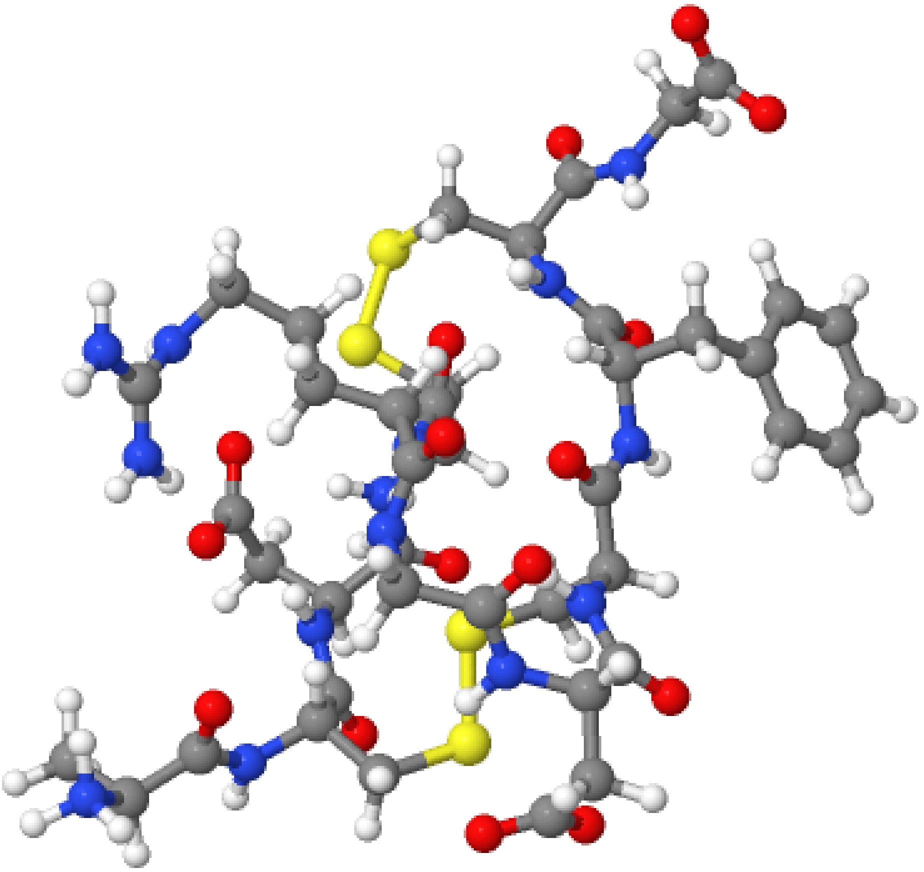

Figure S5: 1FUL

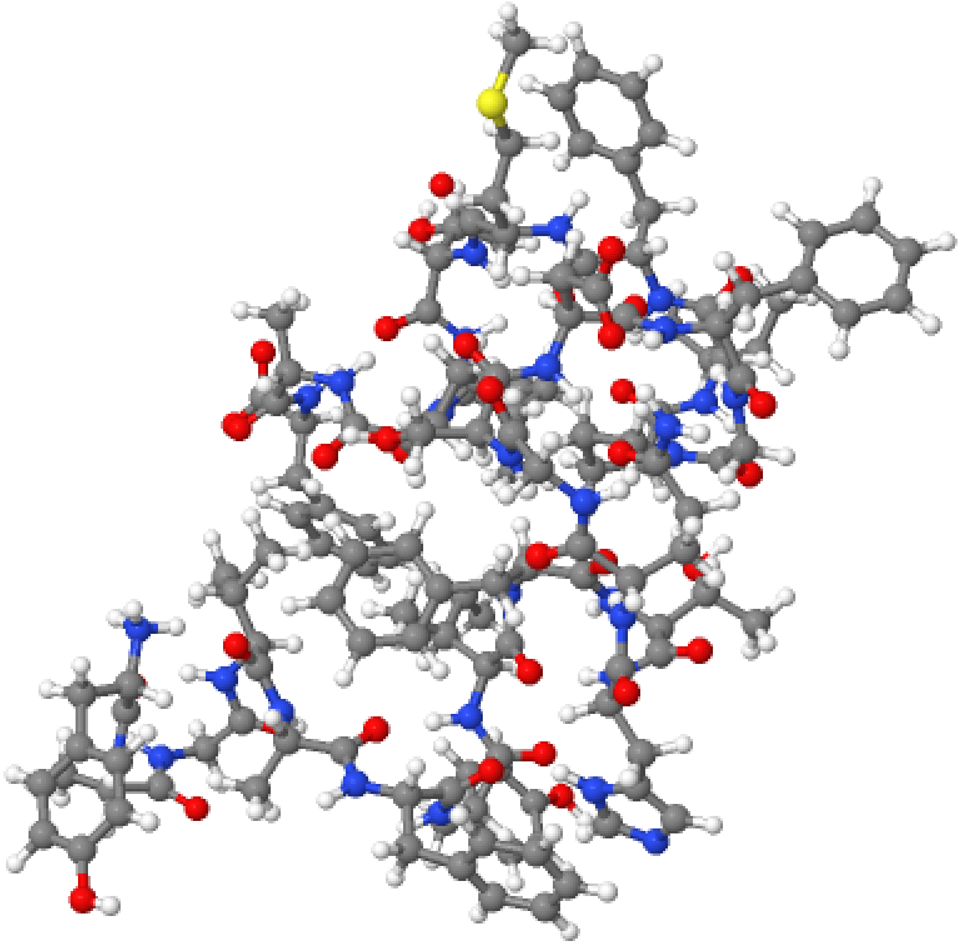

Figure S6: 1EDW

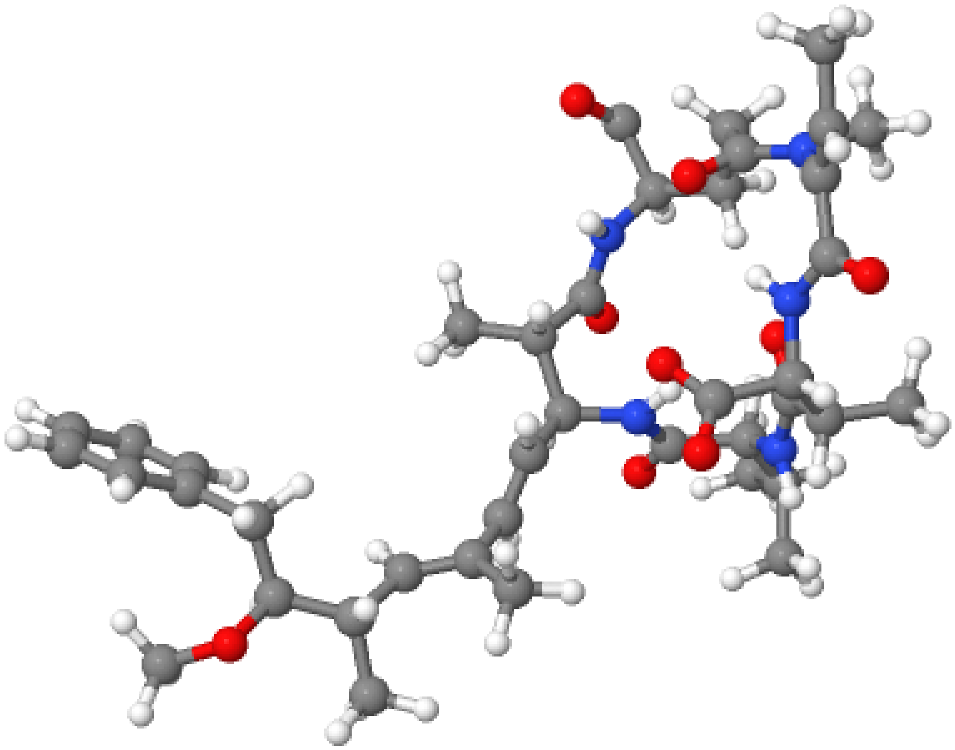

Figure S7: 1EVC

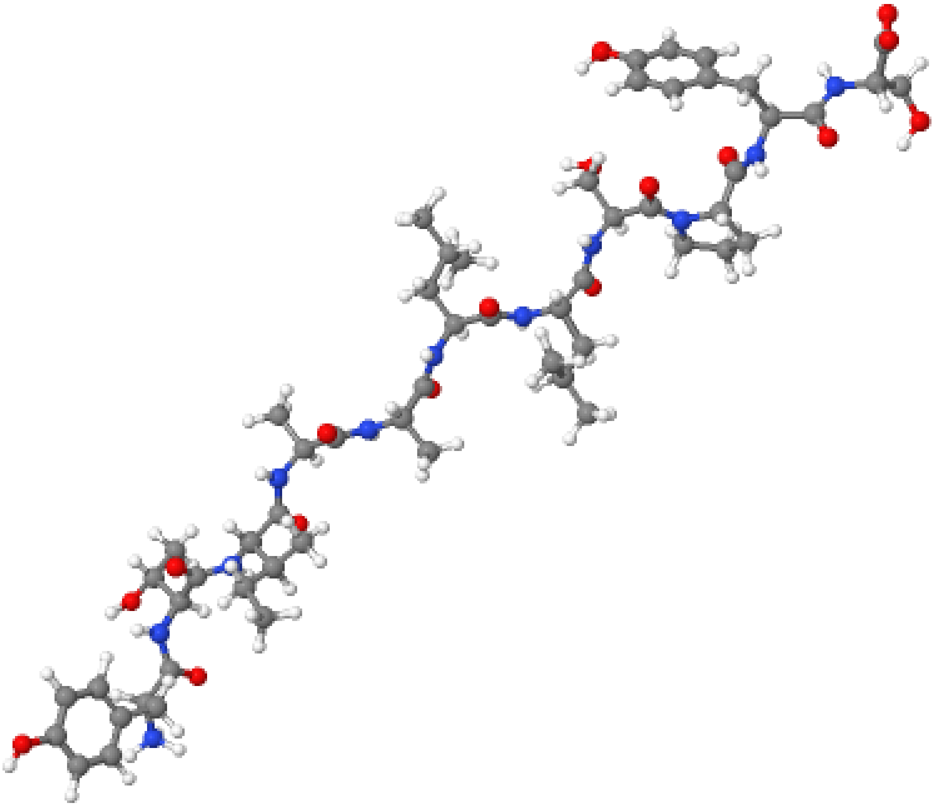

Figure S8: 1RVS

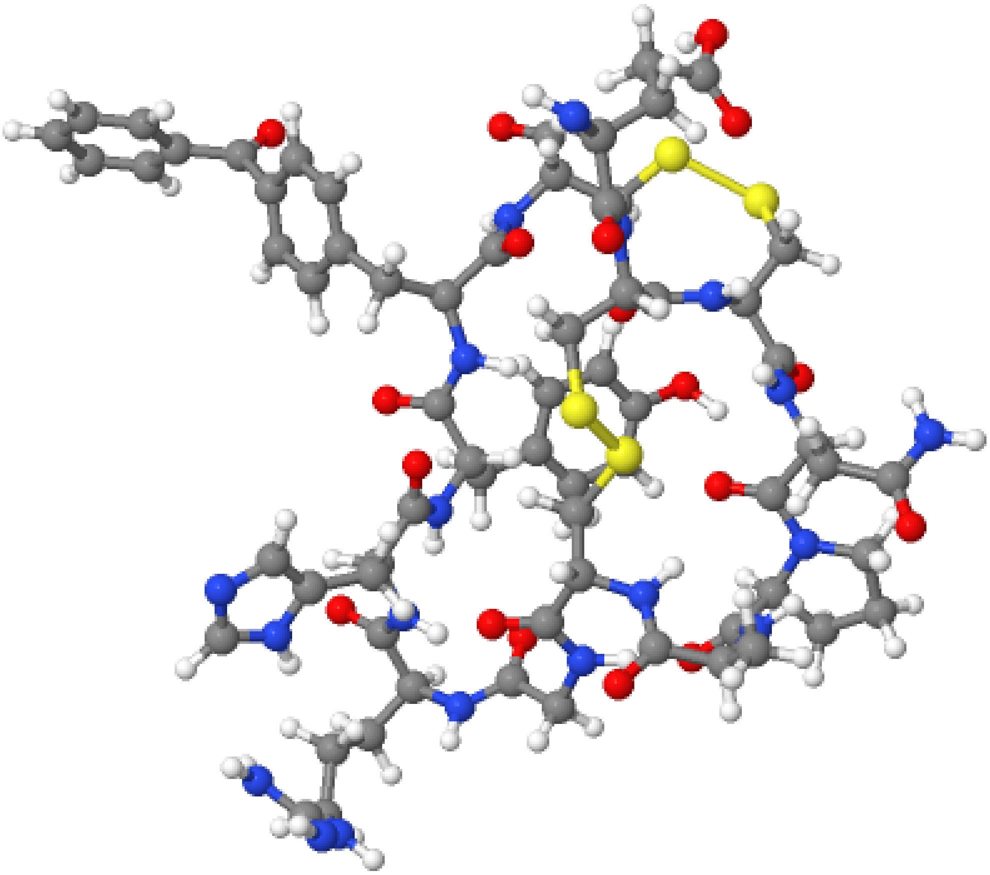

Figure S9: 2FR9

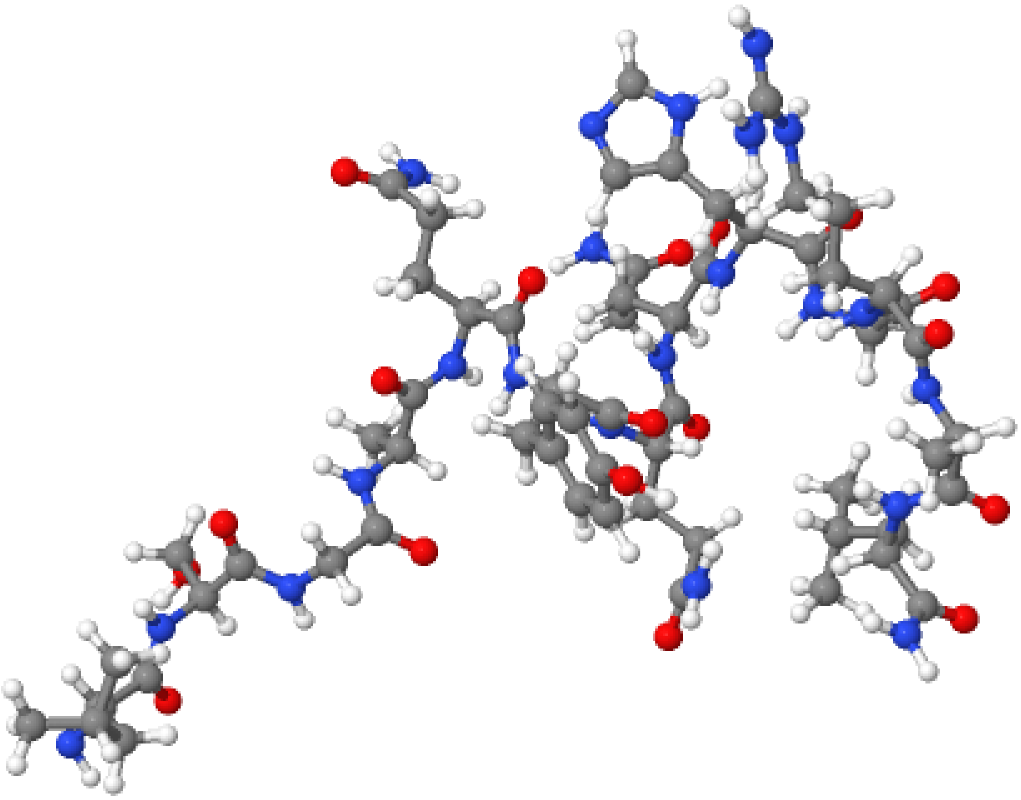

Figure S10: 2JSI

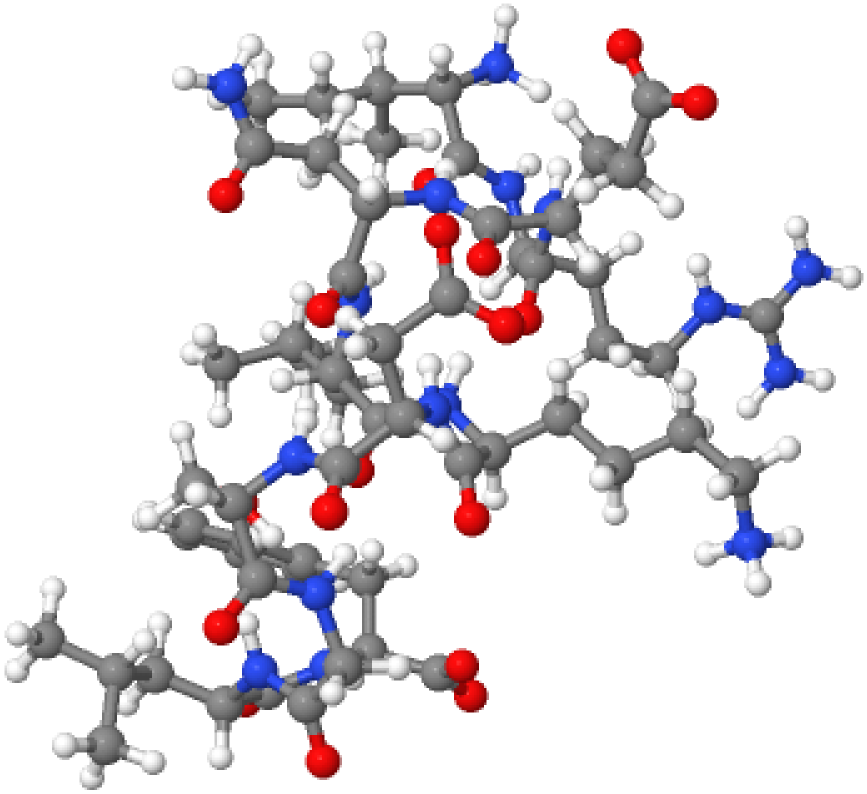

Figure S11: 1LVZ

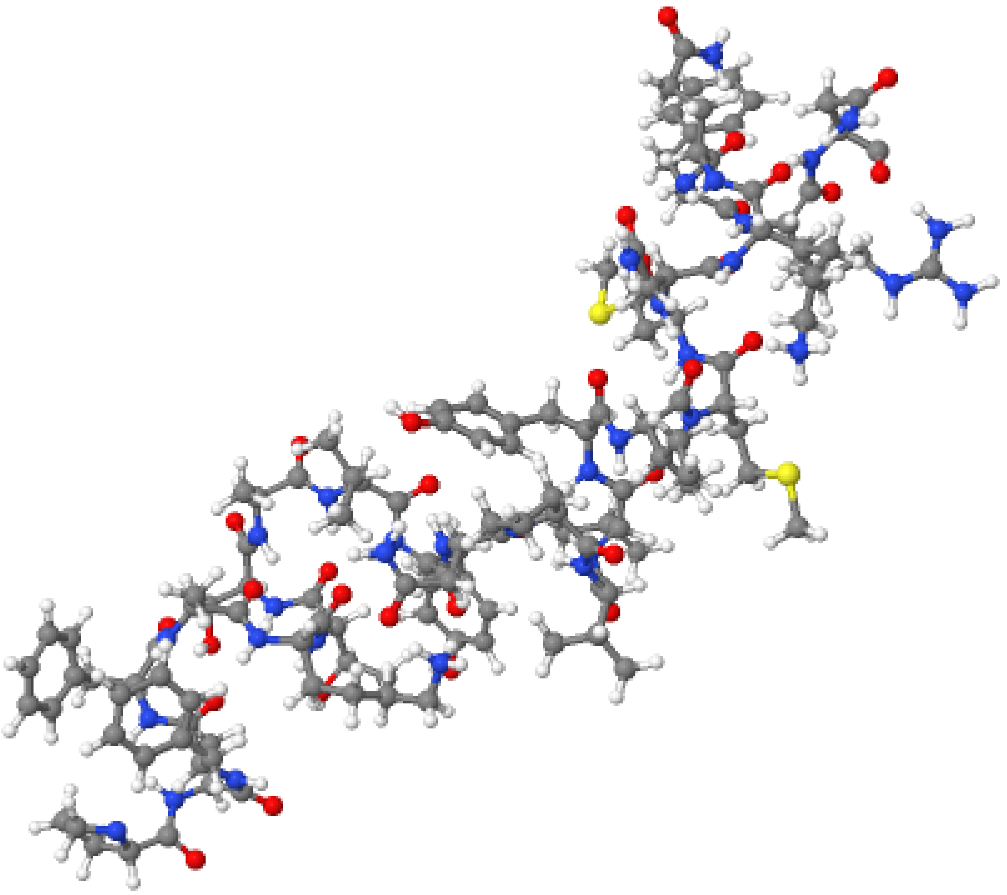

Figure S12: 1FDF

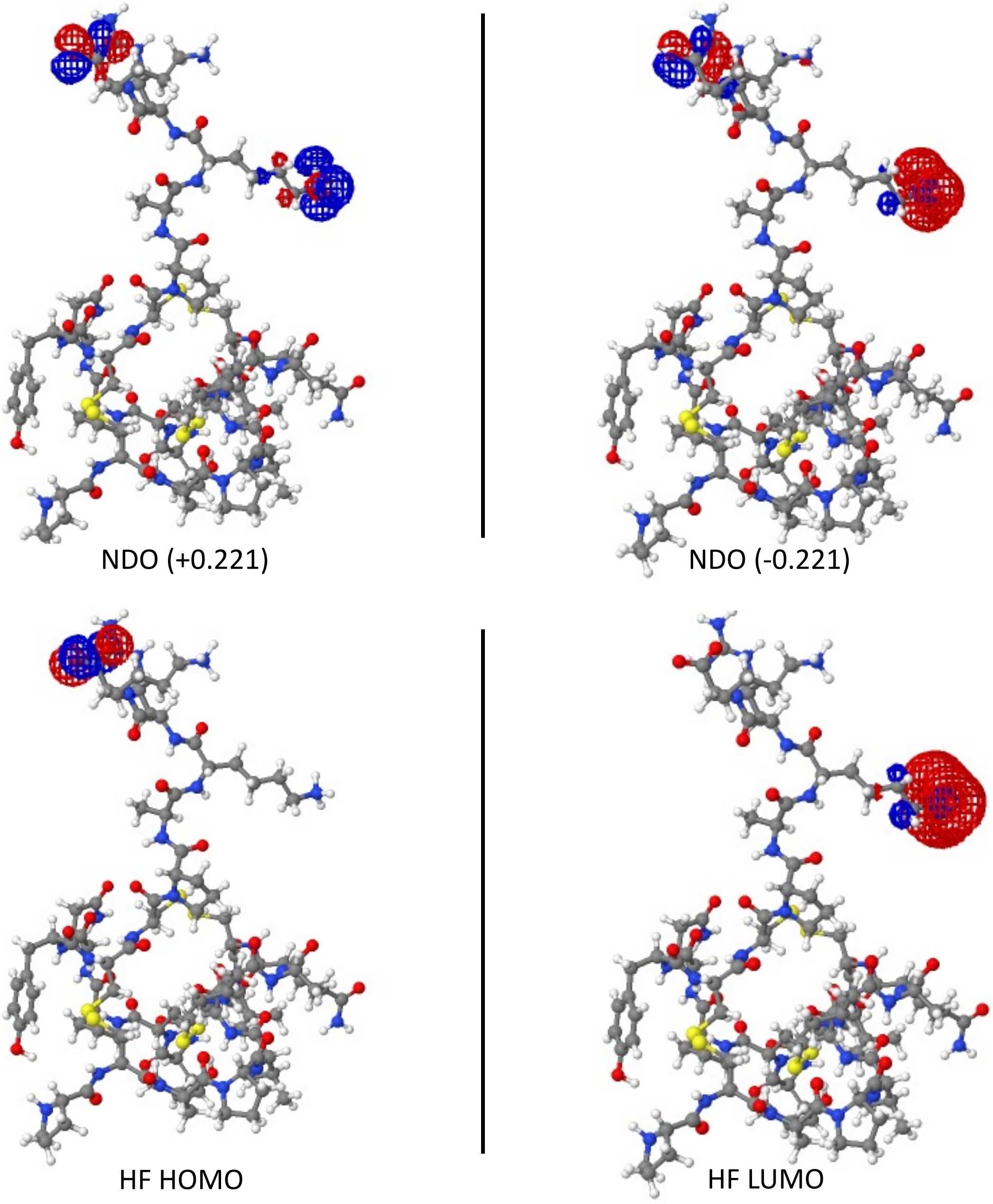

Figure S13: 1SP7: HF-LDA FNDOs, juxtaposed with the Hartree-Fock HOMO and LUMO.

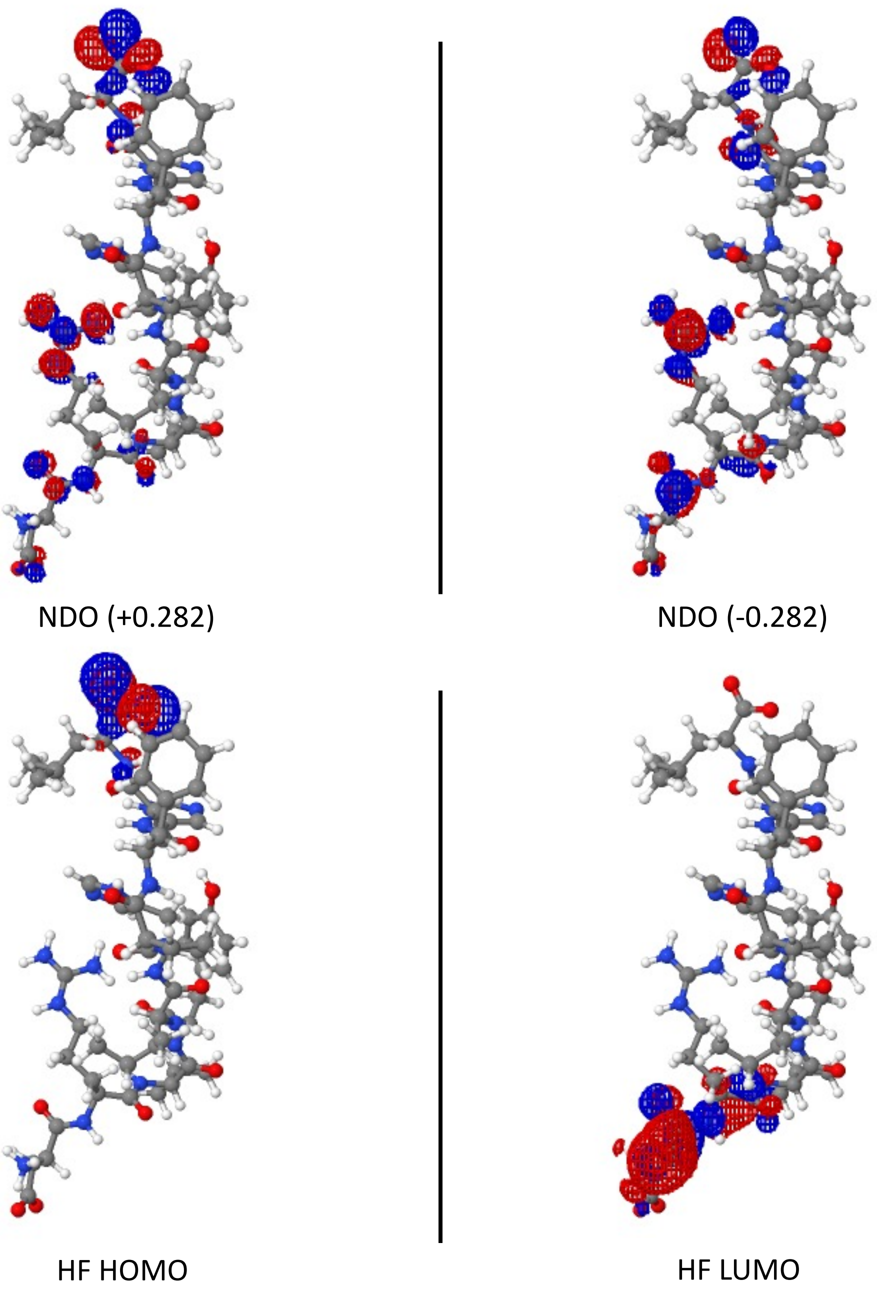

Figure S14: 1N9U: HF-LDA FNDOs, juxtaposed with the Hartree-Fock HOMO and LUMO.

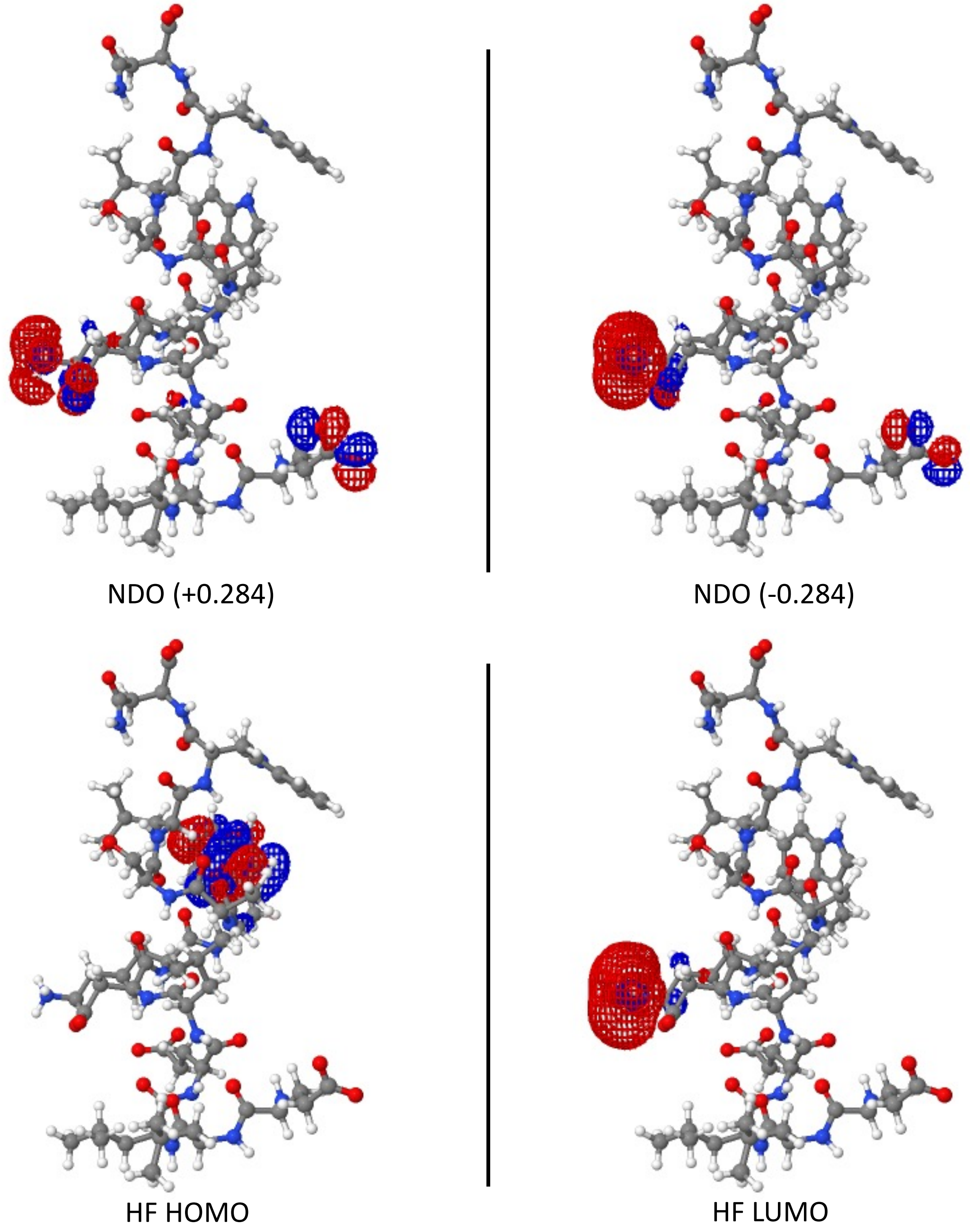

Figure S15: 1MZI: HF-LDA FNDOs, juxtaposed with the Hartree-Fock HOMO and LUMO.

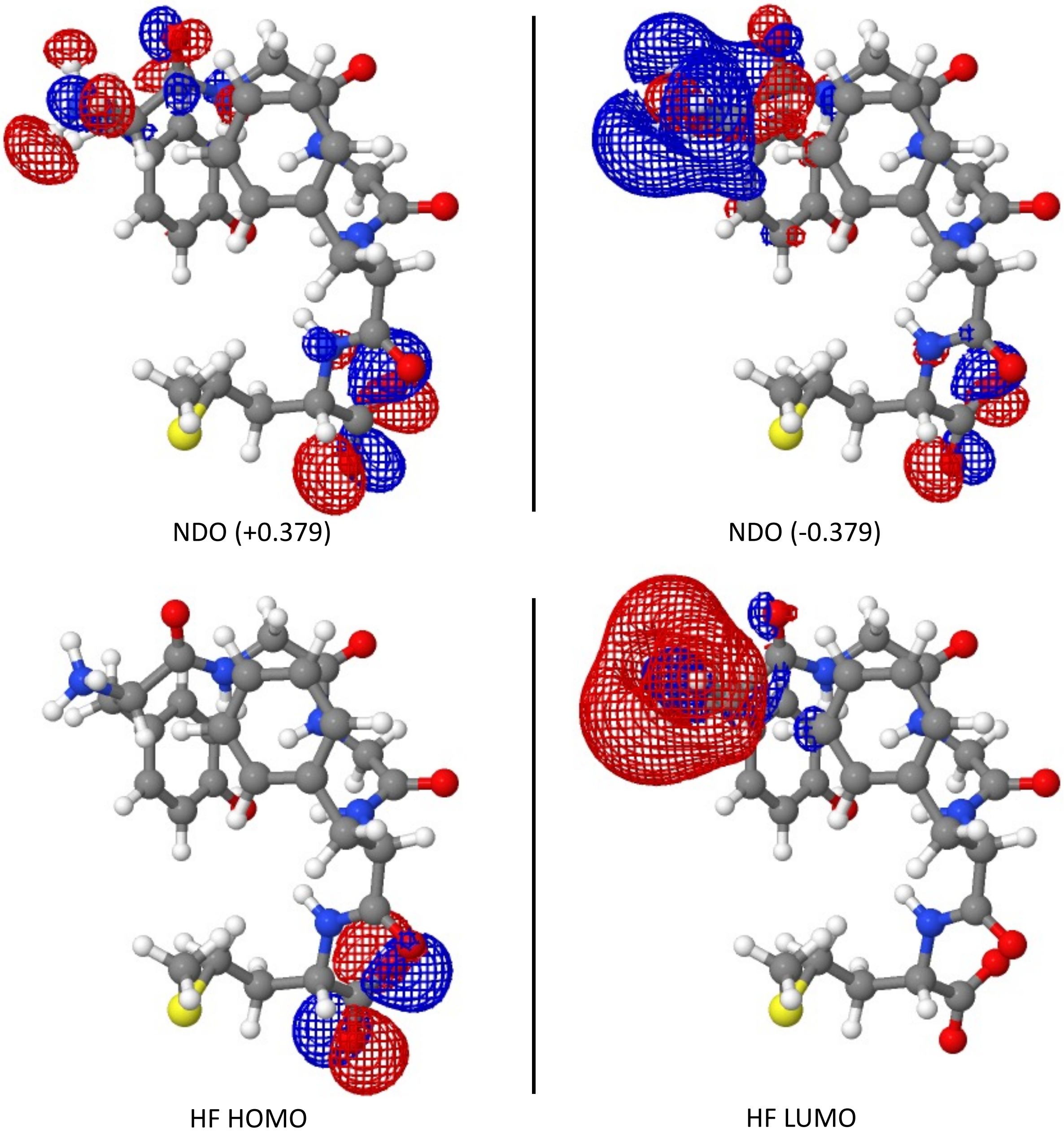

Figure S16: 1PLW: HF-LDA FNDOs, juxtaposed with the Hartree-Fock HOMO and LUMO.

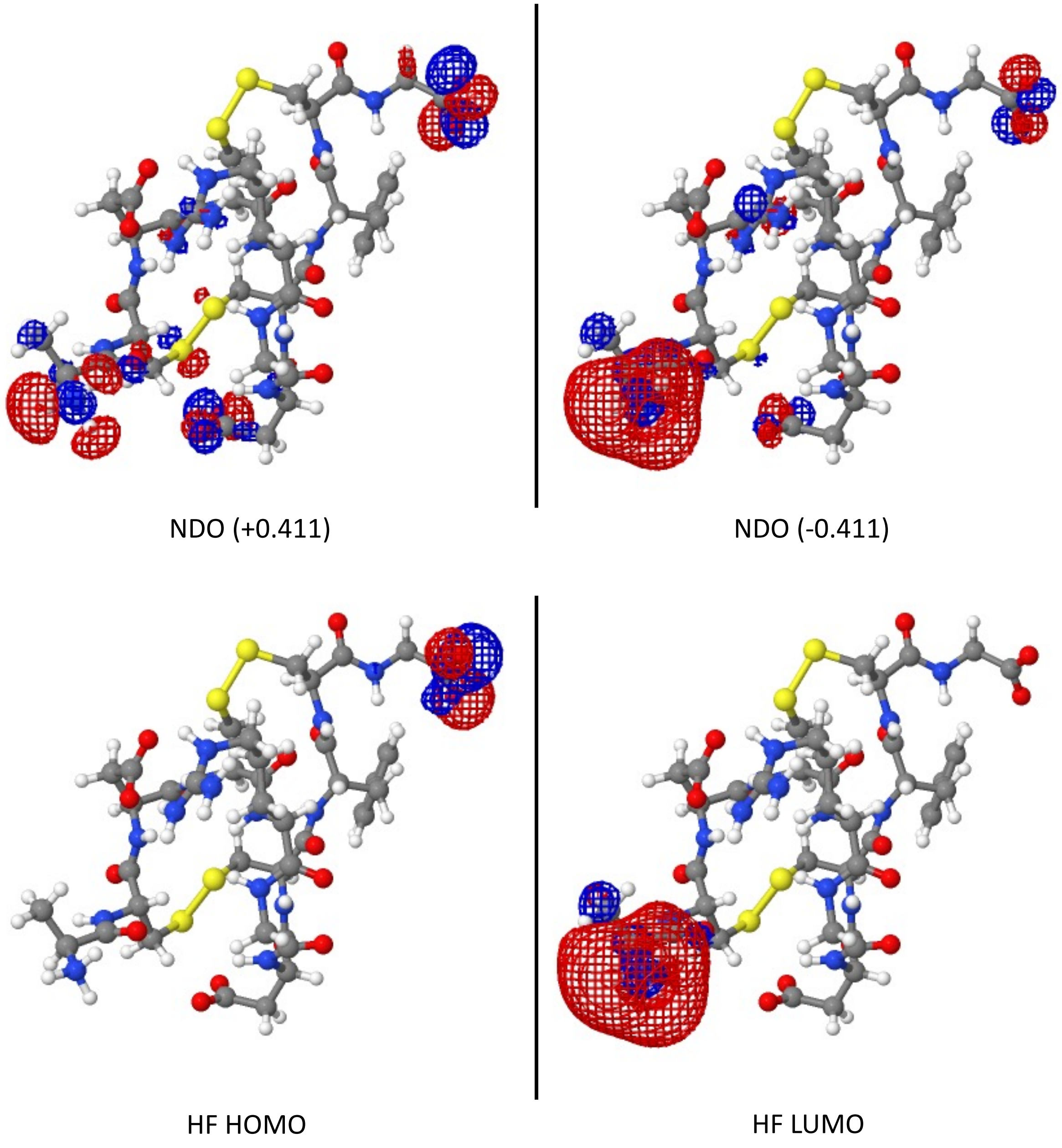

Figure S17: 1FUL: HF-LDA FNDOs, juxtaposed with the Hartree-Fock HOMO and LUMO.

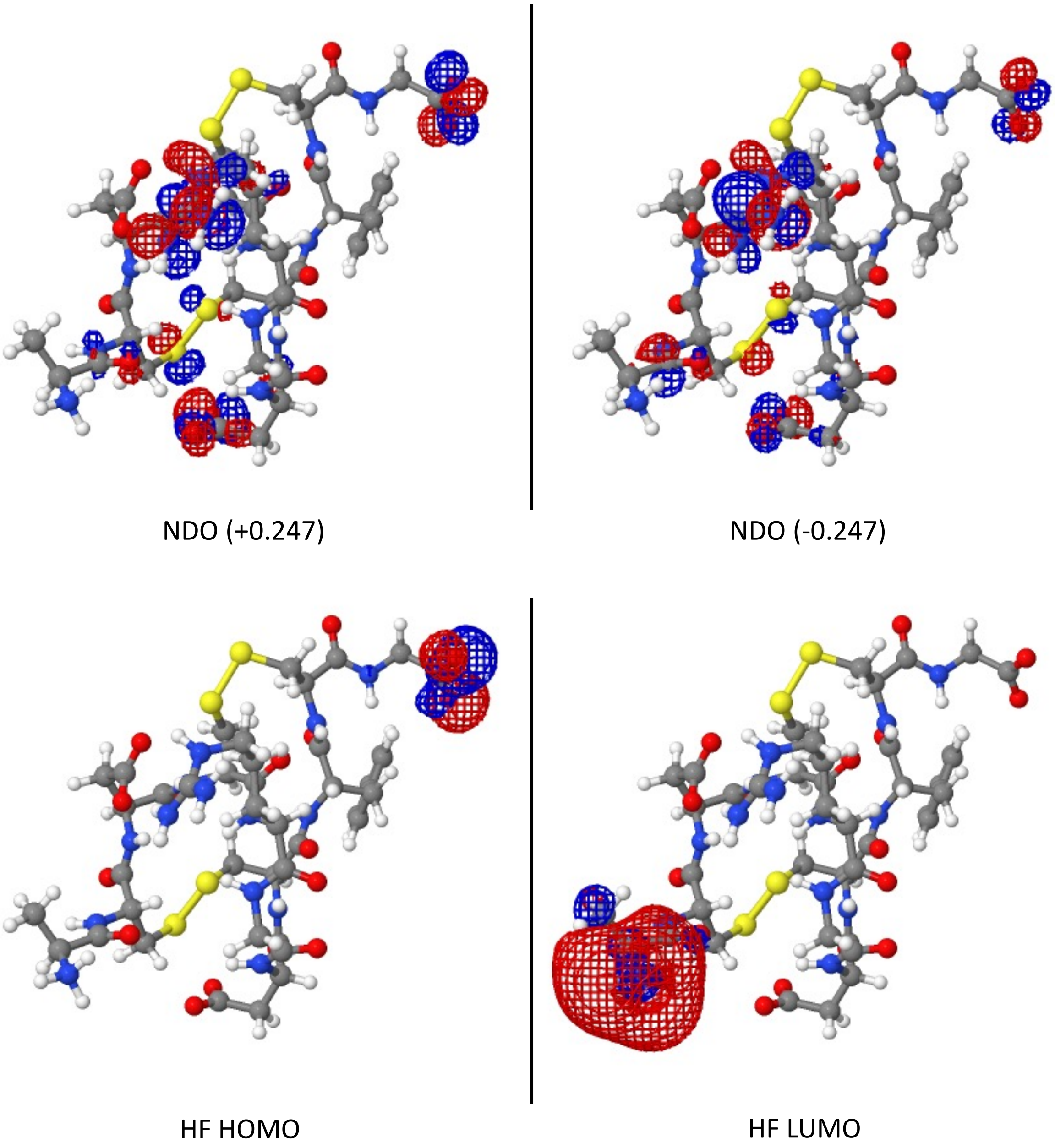

Figure S18: 1FUL: HF-LDA FNDOs, juxtaposed with the Hartree-Fock HOMO and LUMO.

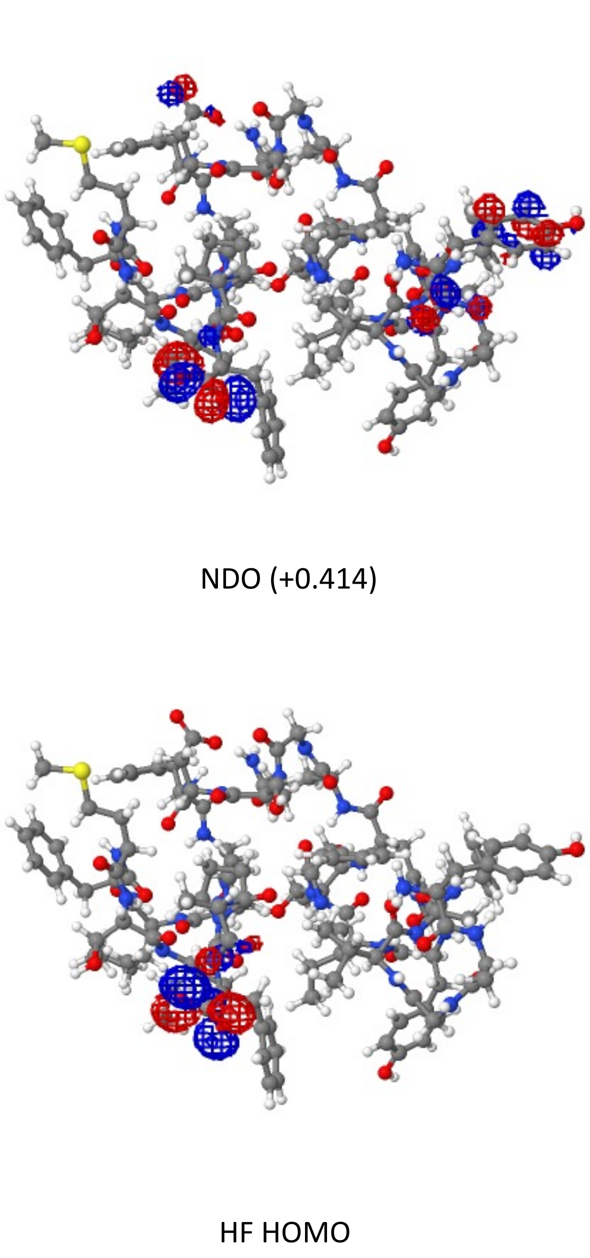

Figure S19: 1EDW: HF-LDA FNDOs, juxtaposed with the Hartree-Fock HOMO and LUMO.

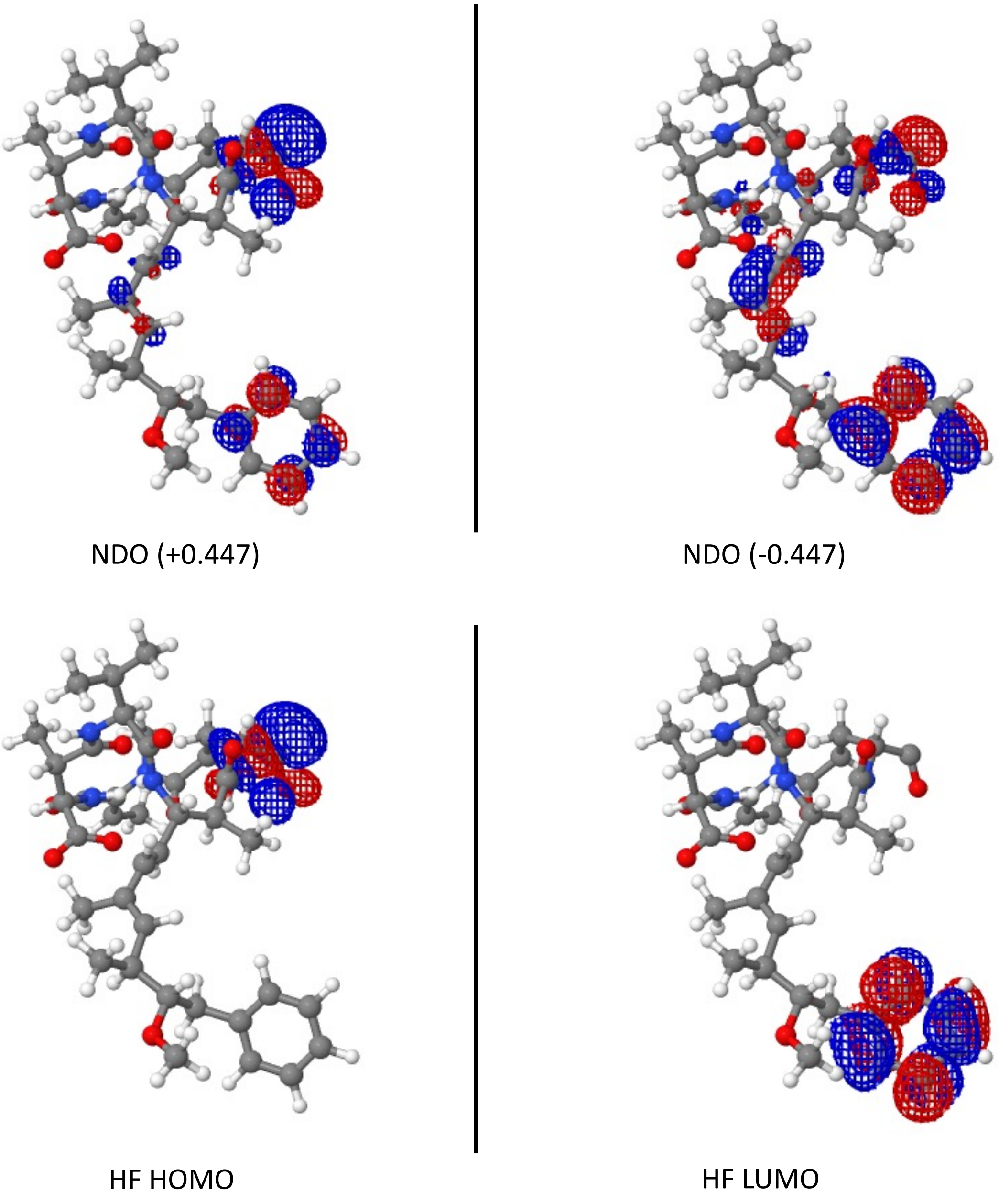

Figure S20: 1EVC: HF-LDA FNDOs, juxtaposed with the Hartree-Fock HOMO and LUMO.

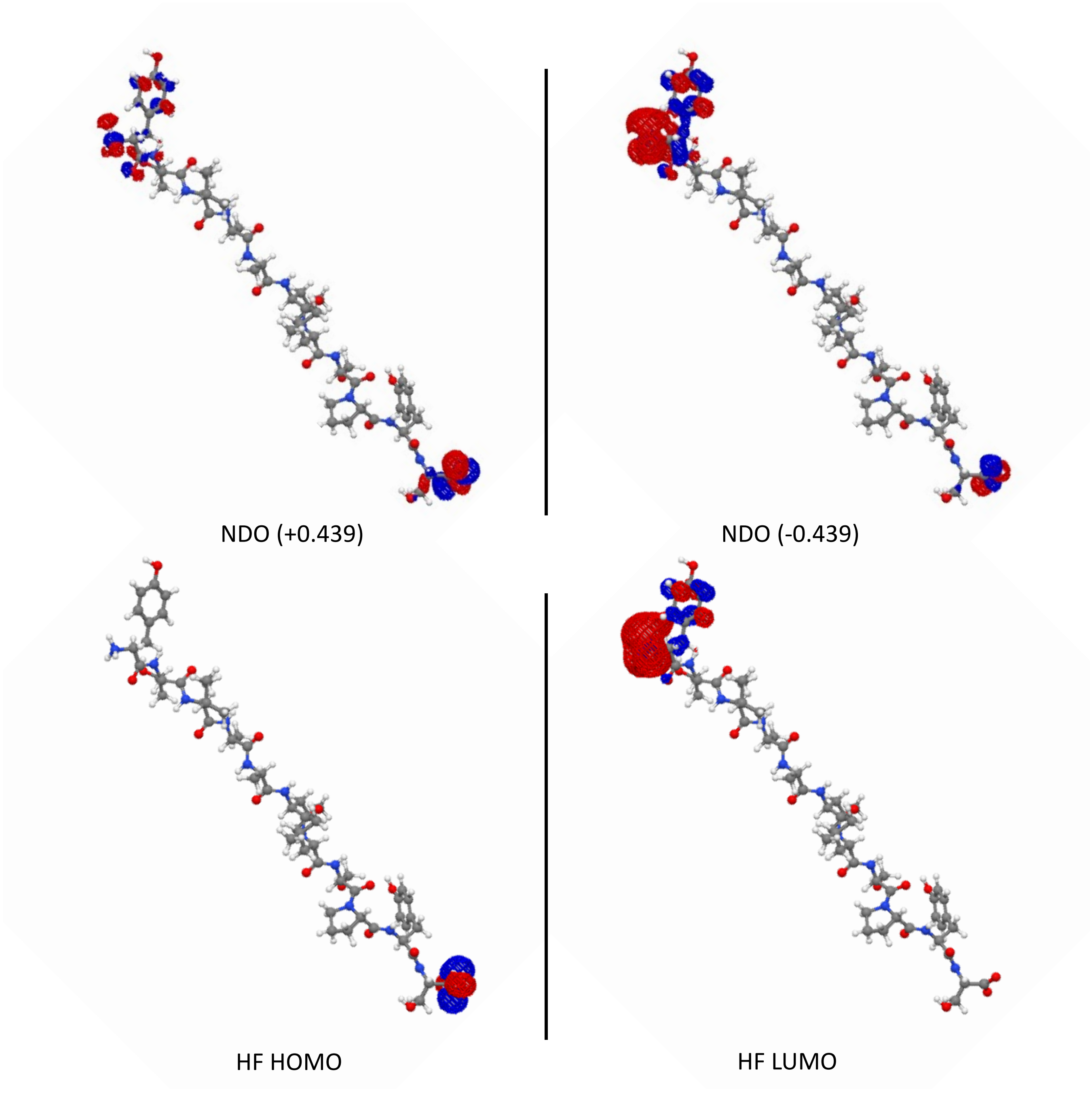

Figure S21: 1RVS: HF-LDA FNDOs, juxtaposed with the Hartree-Fock HOMO and LUMO.

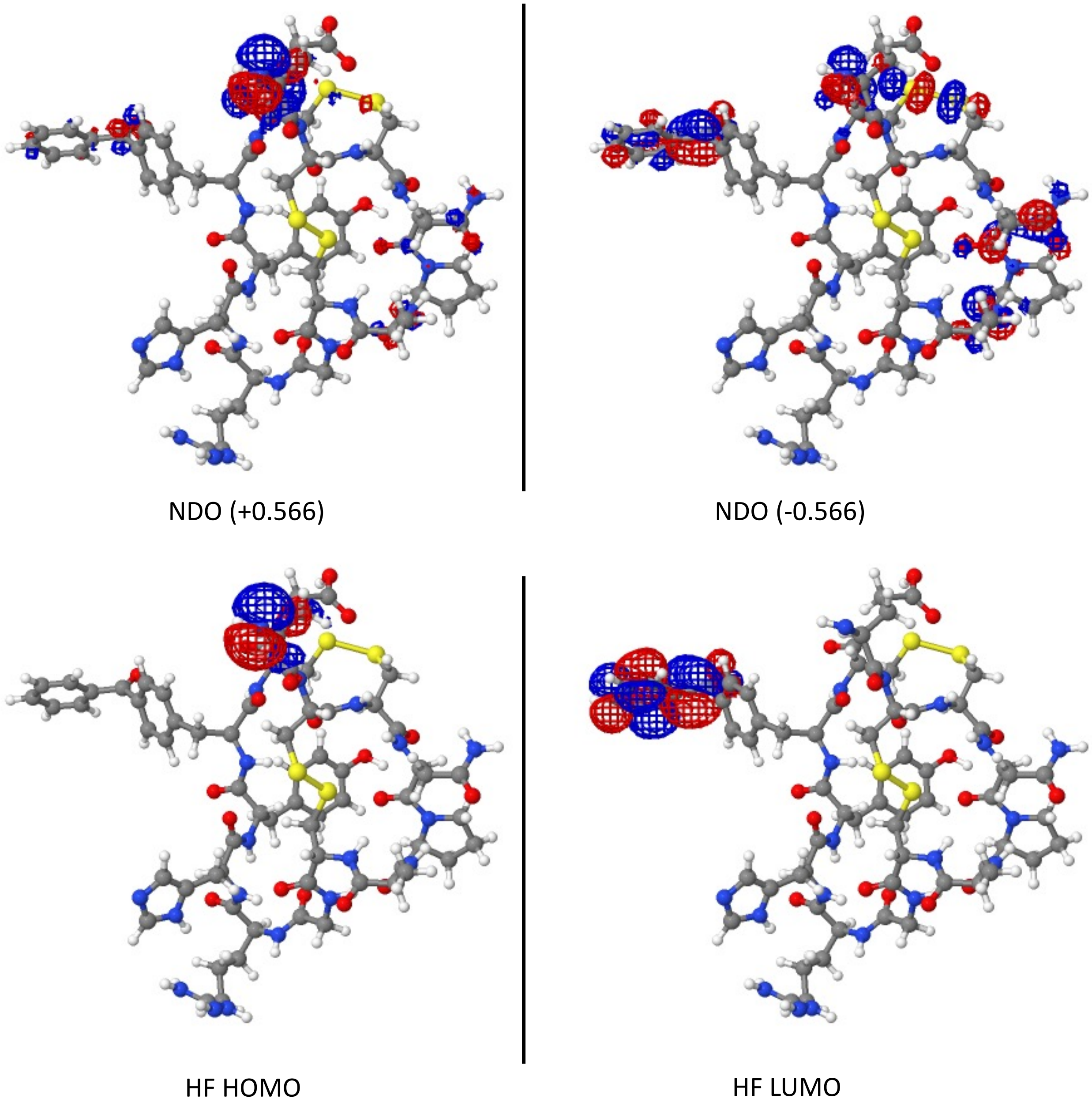

Figure S22: 2FR9: HF-LDA FNDOs, juxtaposed with the Hartree-Fock HOMO and LUMO.

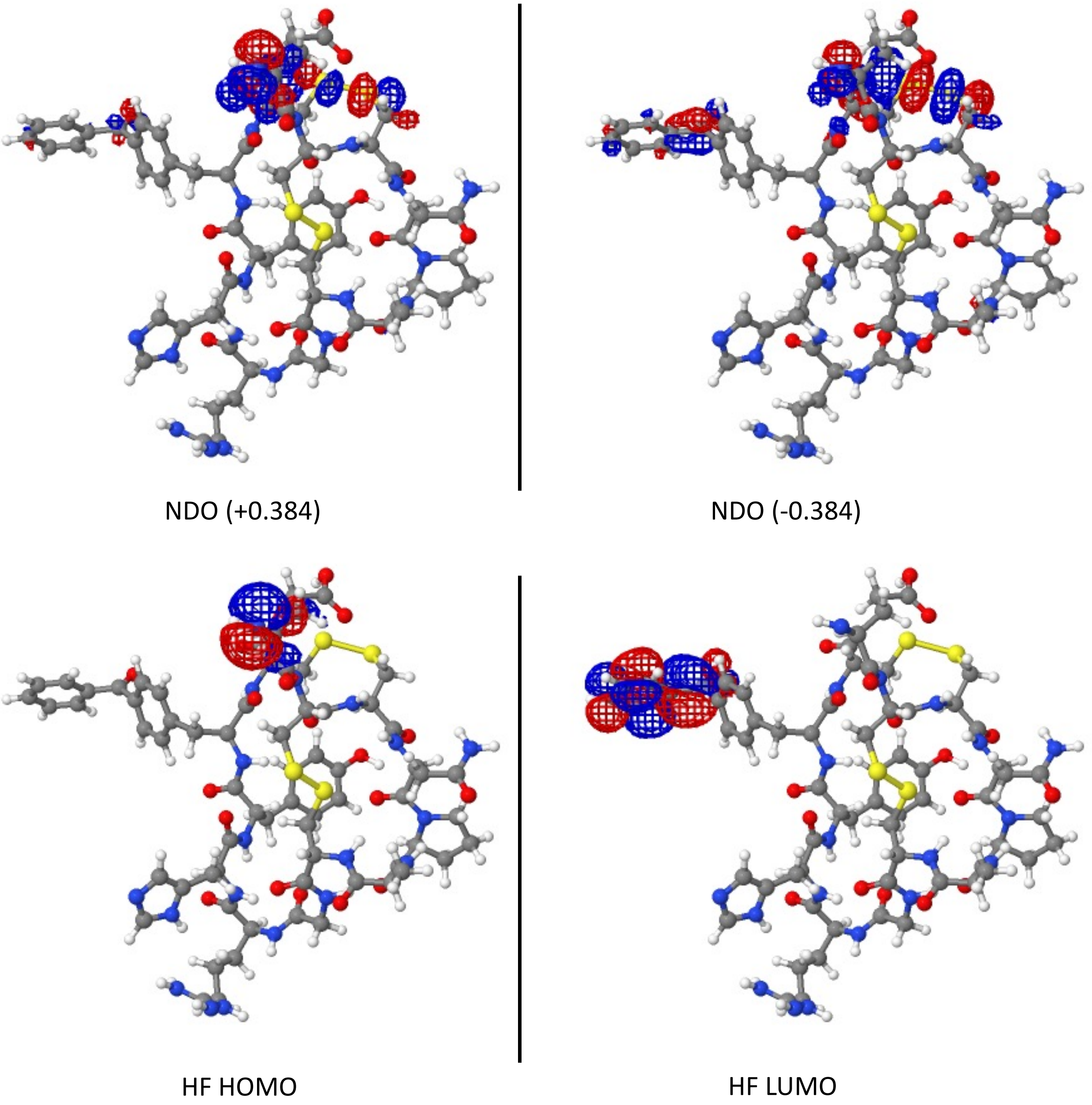

Figure S23: 2FR9: HF-LDA FNDOs, juxtaposed with the Hartree-Fock HOMO and LUMO.

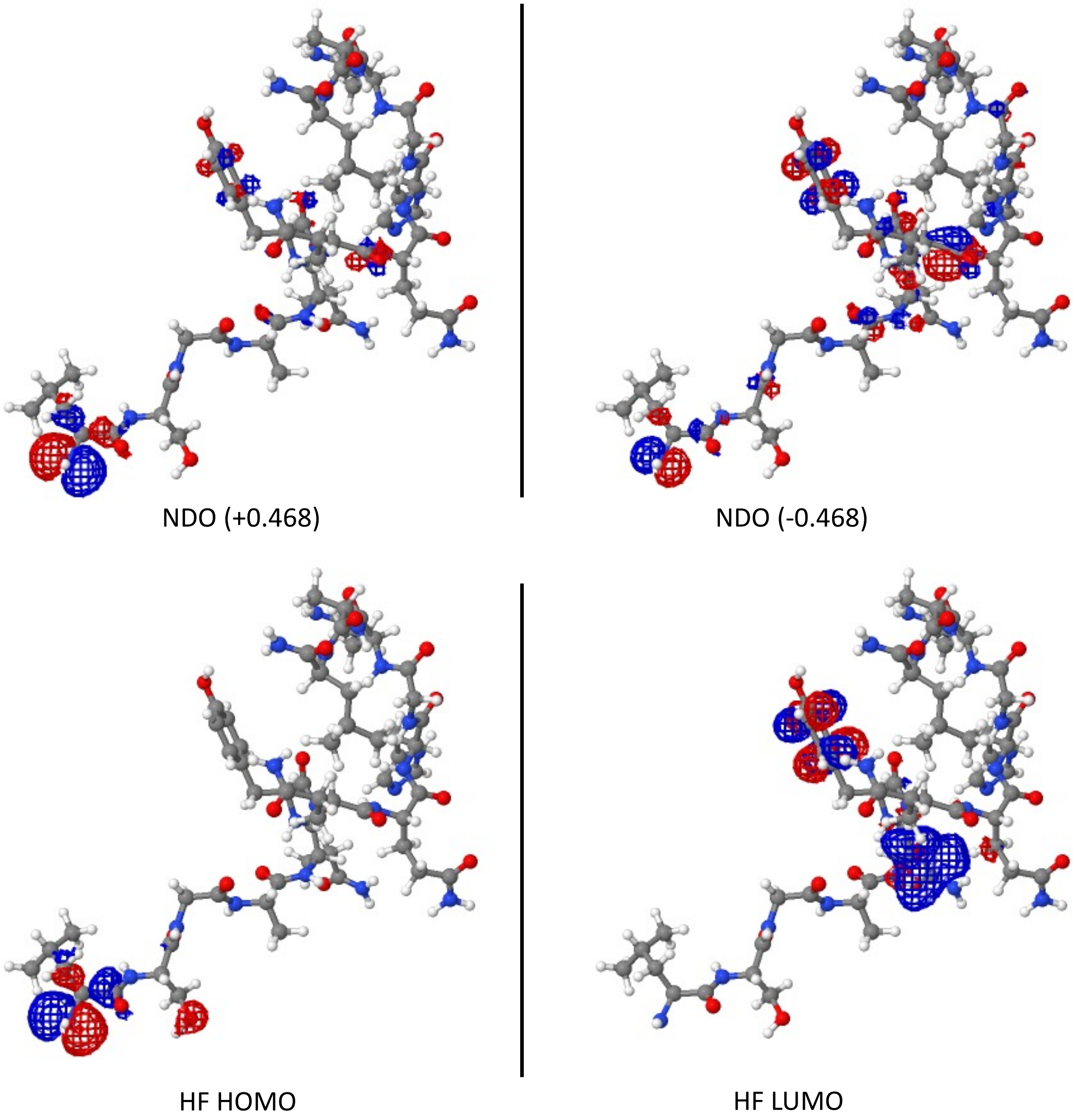

Figure S24: 2JSI: HF-LDA FNDOs, juxtaposed with the Hartree-Fock HOMO and LUMO.

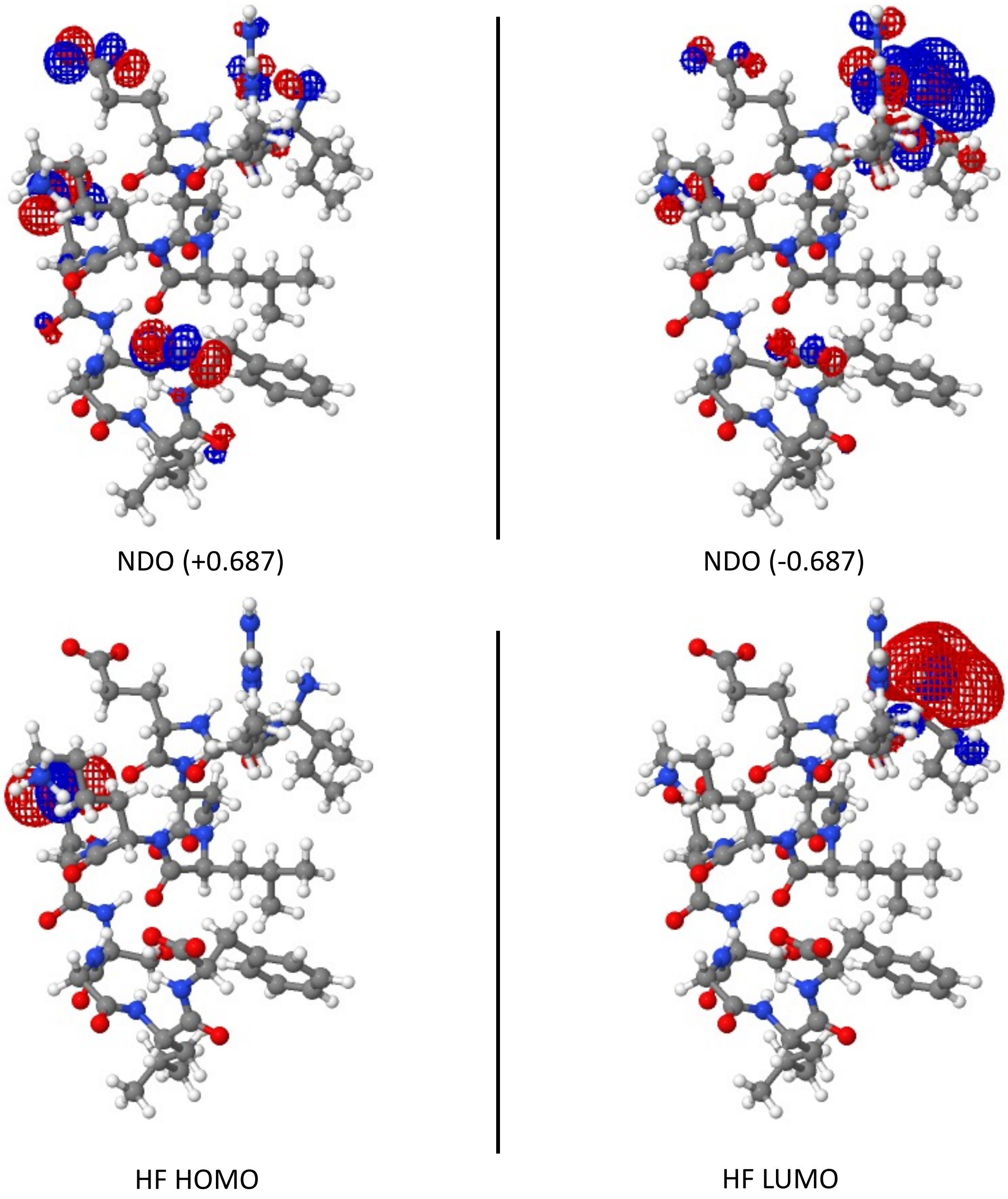

Figure S25: 1LVZ: HF-LDA FNDOs, juxtaposed with the Hartree-Fock HOMO and LUMO.

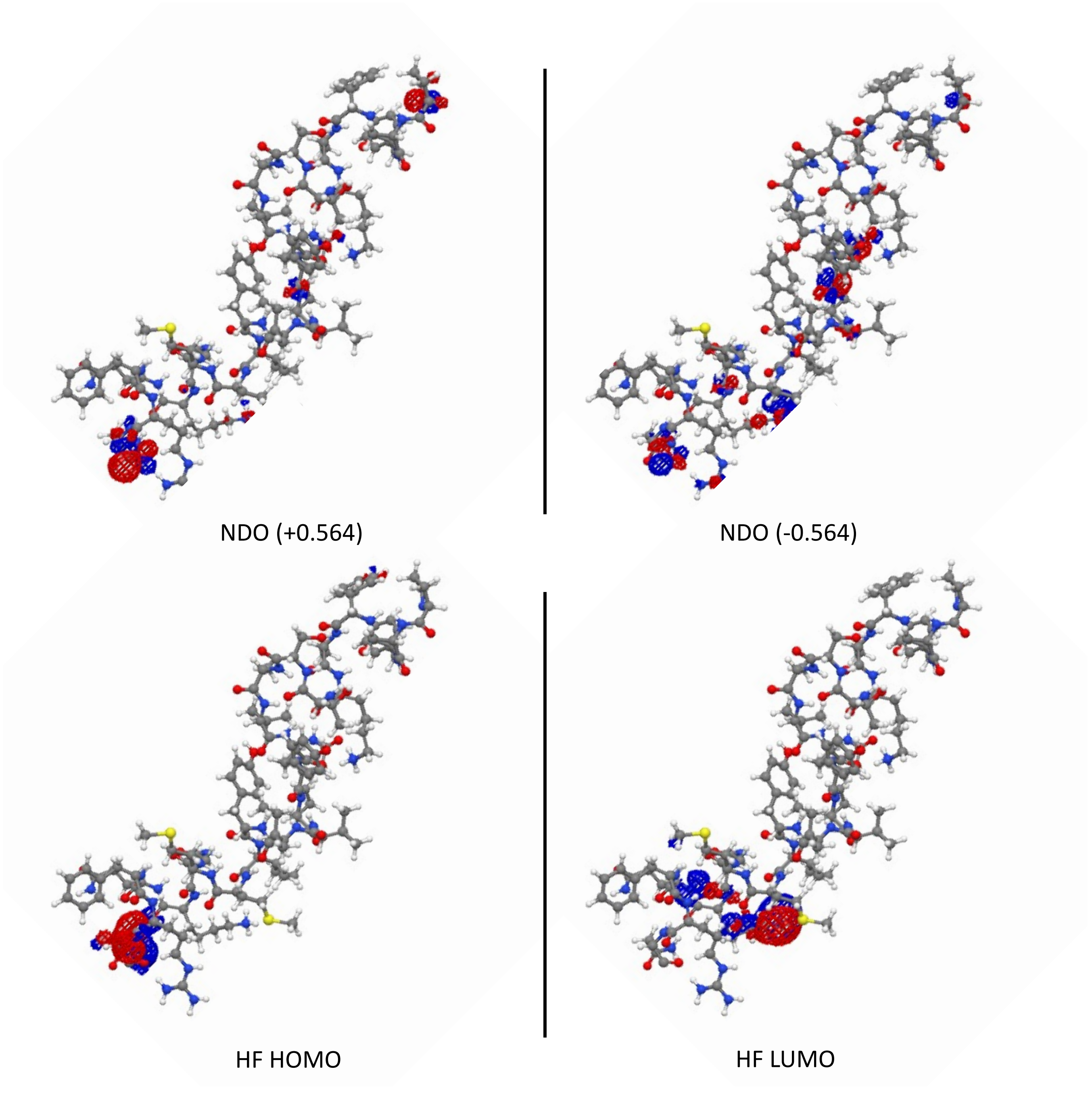

Figure S26: 1FDF: HF-LDA FNDOs, juxtaposed with the Hartree-Fock HOMO and LUMO.

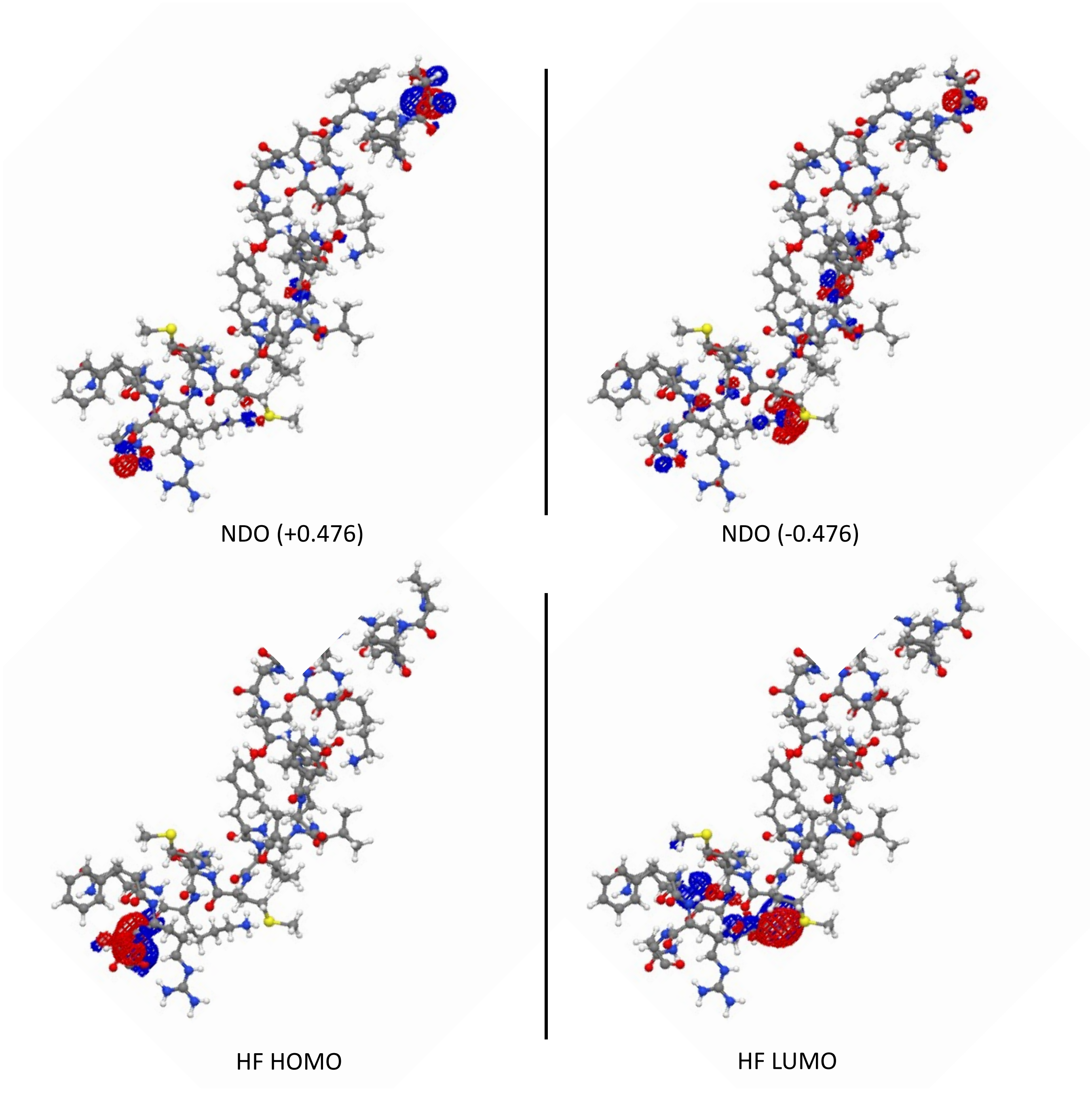

Figure S27: 1FDF: HF-LDA FNDOs, juxtaposed with the Hartree-Fock HOMO and LUMO.
